# Understanding the cultural identity of EFL learners from the eco-linguistics perspective: evidence from students in arts college before and after the COVID-19 epidemic period

**DOI:** 10.3389/fpsyg.2023.1249334

**Published:** 2023-10-05

**Authors:** Yue Peng

**Affiliations:** School of Arts and Humanities, Guangzhou Academy of Fine Arts, Guangzhou, China

**Keywords:** anxiety/uncertainty management theory (AUM), cultural identity, COVID-19, ecolinguistics, identity negotiation theory

## Abstract

The identity development process has individual and societal components and is inherently intertwined with one’s broader sociocultural milieu. The correlation between the personal and social aspects of an individual’s identity considerably influences their behavior within their environment. This study examines cultural identity changes among English as a Foreign Language (EFL) students by conducting a questionnaire survey based on the anxiety/uncertainty management theory by Gudykunst (1995, 1998). The questionnaire was distributed twice: Study 1 used data from 483 students and Study 2 used data from 359 students. After each study, 20 students’ descriptions of Chinese and Western cultures were analyzed in NVivo. Guided by the ecological research paradigm, this study examines the impact of individual differences on cultural identity at the macro-, meso-, exo-, and micro- levels. The findings suggest that social context can influence an individual’s cultural identity, and cultural identity development accompanies being “oneself.”

## 1. Introduction

Identity formation occurs in contexts that are affected by numerous elements. The relationships between language, culture, and identity should be studied in a manner that takes into account the complexities of defining long immersions and delineating boundaries in an infinite intercultural context. Schecter (2014) reviewed three perspectives of researching identity and language: “social anthropology, sociocultural, and participation/relational.” These approaches examine how social boundaries are upheld, how individuals and groups achieve self-sufficiency and success, and how individuals contextualize identities within their actions, speech, and social connections. Progression from rudimentary cultural identification to intercultural identification is a significant avenue for fostering cross-cultural communicative proficiency. Kim’s (2001) proposition of cross-cultural identity offers a valuable framework for constructing one’s cultural self-identity. Kim’s (2001) concept of cross-cultural identity is not limited to a dichotomous approach that incorporates local and foreign cultures. Instead, it encompasses an identity through which one perceives oneself as a global citizen (Sussman, 2000). This viewpoint allows a broader and more accepting understanding of one’s own identity and foreign cultures (Kim, 2017b,c).

From an eco-linguistic perspective, an individual’s cultural identity “emerges” from dynamic cultural interactions. Stibbe (2021) applied “ecological identity” to explain that the identity issue in eco-linguistics is beyond the boundaries of cultures because “ecological identity” is “a sense of belonging to a group which includes not only humans but also others from the wider community of life.” The degree to which a specific community employs a separate array of resources (modes) in its day-to-day social interactions is positively correlated with the level of proficiency attained by its constituents. Furthermore, as Norton and De Costa (2018) stated, the scale research approach to identity investigates how language learners’ backgrounds influence their commitment to language acquisition. To better understand the social reality that has significant effect on language acquisition, considering both temporal and spatial scales is helpful. We use a multi-scalar approach to consider different levels of cultural identity and select English as a Foreign Language (EFL) learners as the research participants because they can provide valuable insights into how cultural differences are navigated when learning a foreign language. Kozaki and Ross (2011) discussed various cases of EFL learners in Japan and Korea and demonstrated that foreign and native languages frequently influence cultural identity conflicts. This indicates that from the perspective of eco-linguistics, cultural identity encompasses the nature of blending timescales, spaces, experiences, and cognition processes. If the story of identity is examined by its unique rather than universal features, the elements that shape identity should be explored through dynamic intercultural interactions.

Cultural identity is not isolated from time and space as it is the culture itself. To avoid investigating cultures and languages in essentialist or structuralist manners, the “super-diversity” proposed by Steffensen and Kramsch (2017) and “diversities” proposed by Dervin (2016) are suitable frameworks for analyzing cultural identity. This study aims to examine the attributes of personal cultural identity and the variables that impact their intricacy through the lens of eco-linguistics. Identity construction is a process of self-reflection, adaptation, and correct comprehension of cultural identity security. Successful cross-cultural communication requires individuals to maintain optimal levels of cultural identity security by managing their anxiety and uncertainty. Therefore, to closely study cultural identities, the participants in the qualitative part of this research were selected using Gudykunst’s (1993, 1995, 1998, 2005) anxiety/uncertainty management theory (AUM) and Ting-Toomey’s (2015, 2017) identity negotiation theory. This research focuses on EFL learners who exhibit an ideal level of cultural identity anxiety, and adopts a multi-scale approach from an eco-linguistic perspective to analyze the differentiation and diversity of cultural identity. This study examines the characteristics of their cultural identity and investigates the factors that influence it at the macro-, meso-, exo-, and micro- levels. By exploring the dynamic map of individual cultural identity before and after the COVID-19 pandemic from an eco-linguistic perspective, this study attempts to provide a comprehensive understanding of EFL learners’ cultural identity development paths.

## 2. Literature review

### 2.1. Cultural identity and language

Cultural identity is a broad concept that is composed of “national, racial, ethnic, class-related, gender-based, political, and religious identities (Kim, 2017a).” As Kim (2017b,c) stated, individuals possess diverse cultural identities depending on contextual and temporal factors. Kim (2017b,c) employs the view of “dynamic cultural identity” from the “processual” view of cultures. According to Qumrul Hasan Chowdhury (2016), the perception of culture can be approached from two distinct perspectives: “essentialized” and “processual.” The “essentialized” view considers culture a static, completed, and inherited consequence shared among a particular group of people. In contrast, the “processual” view conceptualizes culture as a constantly developing, adaptable, and emerging entity. Based on the processual view of cultures, the concept of cultural identity can apply Hall’s (1996) standpoint that cultural identity encompasses both the process of “becoming” and the state of “being.” According to Hall (1996), the development of identity depends on the interaction of various power dynamics that facilitate its advancement, evolution, and enrichment within a multicultural environment. From this viewpoint, identity is defined by its inherent instability and susceptibility to change. Dervin (2016) posited that identity is more closely associated with the construction process than with the solid substance of identity. Furthermore, the current proliferation of the Internet has significantly changed the construction of cultural identity. The process of cultural identity formation continues, regardless of whether one is in a foreign country.

As cultural identity formation can be considered a process, language is an important tool with which individuals can identify themselves. Through object language, individuals recognize their connections to the world and construct unique identities through the acquisition and application of language (Torop, 2014). Torop (2014) expanded upon the connection between language and culture using cultural semiotics, which interprets language in communication into a modeling system, including a sign system (myth, literature, poetry), metalanguage (criticism and history of art, music, dance, cinema), and analog (the language of film, dance, music, painting). Language, regardless of whether verbal or non-verbal, is essential to building and creating cultural identity; the more descriptive languages there are in a culture, the more possibilities for self-identification and the constitution of cultural identity (Torop, 2014). Owing to this close relationship between cultures and languages, the development of cultural identity in individuals learning a new language depends on their ability to remain open and responsive to foreign cultures while also maintaining their own cultural identity (Kim, 2015, 2017a,b).

Furthermore, another famous theory about identity in language acquisition is the well-known investment model from Norton (1995, 2013). According to Norton (2013), identity is “how a person understands his or her relationship with the world, how that relationship is structured across time and space, and how the person understands possibilities for the future.” Darvin and Norton (2015) proposed an extended framework for investment that takes place at the intersection of identity, capital, and ideology. This model illustrates the circulation of power within society and how it shapes identity patterns of inclusion and exclusion through language. However, according to Darvin and Norton’s (2021) further research, although the terms “investment” and “capital” highlight the way language functions within a political economy and dynamic powers, the investment model is never static or fixed. The individualized lens for language learning is necessary because learners are “complex beings shaped by dispositions and desires, performing diverse selves and identities while negotiating differences and inequalities (Darvin and Norton, 2021).”

The relationship between cultural identities and languages should not be limited to language learning activities. Schecter (2014) suggested that research on the connection between language and identity should be more global as digital technology is increasingly integrated into daily life. Contemporary studies of identity and language can be divided into two types. The first is outside context effects, such as intercultural adaptation (Lapresta and Huguet, 2008; Soldatova and Geer, 2013; Lou, 2021) and classroom teaching (Català, 2015; Lynch and Motha, 2023; Sang, 2023). The second type is about different forms of identity, such as teacher identity (Edwards and Burns, 2016; Nordstrom, 2020; Chao, 2022; Robertson and Yazan, 2022; Zhang and Hwang, 2023) and professional identity (Skjeggestad et al., 2017; Bagiyan et al., 2021; Desselle et al., 2023). Besides, the research on cultural identity and languages mainly focuses on the issue of national identity (Caldas and Caron-Caldas, 1999; Lai, 2011) and immigrant identity (Petreñas et al., 2018). Other researchers have explored the connection between cultural identity and language in multilingual issues (Hoffmann, 1995; Kung, 2016; Macias, 2023). For English acquisition, studies have primarily concentrated on language teaching (Downes, 2001), language proficiency (Peng and Patterson, 2022), and identity negotiations between one’s native language and English (Gao, 2021). However, in a dynamic cultural context, analysis without the perspective of eco-surroundings is insufficient. According to Clément and Norton (2021), the worldwide dissemination of English has resulted in the disregard of socially derived factors, such as ethnolinguistic vitality and intergroup context, which were not initially relevant to the cultural foundation of English. Therefore, language and identity research must incorporate contextual and intra-psychological processes.

### 2.2. Identity and eco-linguistics

Eco-linguistics, or Language Ecology, is an emerging interdisciplinary area that reveals the interaction between language and ecology by studying the ecological factors of language. Eco-linguistic inquiry pertains to the function of language in fostering life-sustaining engagement with fellow humans, living beings, and natural surroundings (Leather and Dam, 2003; Stibbe, 2021). Haugen (2001) initially defined it as “the study of interactions between any given language and its environment.” According to Haugen (2001), the surrounding language initially directs a person’s cognitive processes toward the referential domain of language indices. Eco-linguistics emphasizes that language research must be closely linked to various external environments. Steffensen and Fill (2014) identified four approaches to language ecology: symbolic, natural, sociocultural, and cognitive. Symbolic ecology examines the coexistence of languages or “symbol systems” in a particular region; natural ecology explores the relationship between language and the biological and eco-systemic environment; sociocultural ecology investigates how language is influenced by social and cultural factors that shape the conditions of speakers and speech communities; cognitive ecology examines how language is facilitated by the interplay between biological organisms and their surroundings (Steffensen and Fill, 2014). From the perspective of eco-linguistics, language reflects individuality and thinking perception and exhibits a multi-scalar nature with various levels and dimensions.

The language cognition ecosystem should include both psychological and sociological aspects. Individuals who acquire, employ, and disseminate language are predominantly responsible for determining their ecology (Haugen, 2001). Ecology adopts a holistic approach toward linguistics, considering a language to be studied in “relation to the personal, situational, cultural, and societal factors” (Kramsch and Vork Steffensen, 2008). From this perspective, eco-linguistics examines the contextual characteristics of language and communication, emphasizing unique and specific aspects rather than the general, and concentrating on complexity, chaos, and diversity rather than the universal (Kramsch and Vork Steffensen, 2008). Cultural identity in language acquisition can be viewed as the process of understanding the self that motivates individual’s learning of languages (Darvin and Norton, 2021). According to Kramsch and Vork Steffensen (2008), Norton considered the individual identity in language learning as “given” by the social world. However, in eco-linguistics, identity construction results from interactions between external and internal contexts. Furthermore, Steffensen and Kramsch (2017) stated that studies of identity in eco-linguistics should focus on “super-diversity,” which includes the multi-scalar factors that influence identity construction. Consequently, if the identity in language learning is not “given” but instead “emerges” from the interaction of external and interior environments, as eco-linguistics proposes, EFL and English as Second Language (ESL) learners would have the same likelihood of encountering cultural identity challenges.

The complexity of language is considered to be “naturalized” in the socialization framework by eco-linguistics. Steffensen and Cowley (2021) stated that “a core eco-linguistic concern is pursuing bio-ecological dynamics.” Some scholars apply the distributed language perspective (DLP) to eco-linguistics. DLP regards language as multi-scalar and multi-logical, operating as a first-order system delimited by a second-order system and thereby establishing connections across various temporal scales within the present moment (Li et al., 2020; Steffensen and Cowley, 2021). The DLP also suggests that an ecological paradigm is a framework that investigates how individuals utilize sociocultural resources in a nonlinear and unforeseeable manner (Li et al., 2020). For the connection between context and language, the eco-linguistics framework has been applied in content analysis (Nash and Mühlhäusler, 2014; Norton and Hulme, 2019; Kaushal et al., 2022), discourse analysis (Alexander and Stibbe, 2014; Zhou, 2017; Huang and Zhao, 2021) and metaphors analysis (Döring and Zunino, 2014). For immigrants in an intercultural context, the eco-linguistic framework has been used in studies on multilingualism and language (Pérez, 2015; Farsiu, 2021). In English teaching and learning, the eco-linguistic perspective is also used in content (Zahoor and Janjua, 2020) and discourse (Goulah, 2017) analysis. These studies have mainly examined the relationship between participants’ multi-layered environments and language but rarely focused on the connection between self-identity and language in the ecosystem. According to Stibbe (2021), the perspective of eco-linguistics has the potential to explore how narrative language usage within society can contribute to the formation of identities. From the eco-linguistics angle, self-identity is “an evolving story” that is always in the ongoing process of being constructed, upheld, and modified.

### 2.3. Cultural identity negotiation from an eco-linguistics perspective

The relationship between language and identity exists across diverse cultural contexts. According to English and Marr (2015), the communication process entails the utilization of diverse modes, and the intricate interweaving of these modes results in the meaning production. Identity construction in communication is not passively “given” by one’s surroundings. From an eco-linguistics perspective, cultures, languages, and identities are diversely intertwined; however, not all individuals engaged in intercultural contexts can achieve effective communication. Gudykunst’s anxiety/uncertainty theory (AUM) proposes that an individual’s management of their anxiety and uncertainty directly affects the effectiveness of their cross-cultural communication in interpersonal and intergroup interactions (Gudykunst, 1993, 1995, 1998, 2005; Gudykunst and Nishida, 2001). Individuals can communicate effectively by regulating their anxiety levels and making accurate predictions about others’ attitudes and actions (Gudykunst, 1995). According to Gudykunst (1993, 1995), successful intercultural communication depends on managing anxiety and uncertainty within a specific range falling between the minimum and maximum thresholds. Failure to manage these factors can lead individuals to disengage from interactions and resist further communication (Gudykunst, 1993, 1995; Gudykunst and Nishida, 2001).

Eco-linguistics advocates view identity as naturalized and emerging, while from the standpoint of intercultural communication competence (ICC), identity construction involves active exploration. Identity construction in an intercultural context is intricate and multifaceted, necessitating a certain level of internal conflict between one’s allegiance to one’s original identity and one’s desire to adopt a new one (Kim, 2015). In addition, the notion of “mindfulness” in AUM theory contextualizes that intentionally managing uncertainty and anxiety is essential for successful cross-cultural communication (Gudykunst, 1993, 1995). The emergence of cultural identity within a dynamic ecosystem is possible; however, without effective management, such identity construction cannot endure the shock of cultural diversity. Hence, if the surrounding ecosystem shapes one’s identity, it is imperative to address individuals’ efforts to overcome linguistic and cultural barriers and obstacles during identity formation. Lund and Walston (2020) proposed that library anxiety involves various factors, including beliefs, motivation, expectations, willingness, and uncertainty or anxiety management (AUM). This indicates that anxiety will not occur solely in internal and external contexts. AUM theory can be applied in various communication with external contexts, such as intercultural communication (Hullett and Witte, 2001; Miller and Samp, 2007; Samochowiec and Florack, 2010; Ni and Wang, 2011), professional communication (Logan et al., 2016; Prince, 2021), and communication with strangers (Duronto et al., 2005). For the internal context, the AUM theory has been used in cross-cultural adjustment and adaption (Hua et al., 2019; Pang, 2020), sociocultural adaptation (Sarmiento et al., 2019), acculturation and ideology (Peng et al., 2019), and intercultural competence (Peng et al., 2020). According to the literature, external and internal conditions continuously affect individuals’ uncertainty and anxiety.

Moreover, Ting-Toomey’s (2015, 2017) identity negotiation theory expands AUM theory by introducing the process of creatively managing and coordinating one’s cultural identity in cross-cultural communication. Similar to AUM theory, identity negotiation theory posits that there exists an optimal threshold range for security in diverse identity negotiation scenarios (Ting-Toomey, 2015, 2017). Ting-Toomey (2015) stated that individuals feel anxious in unfamiliar cultures and more secure in familiar cultures despite the fact that everyone desires identity security. Appropriate security can produce good identity negotiation results and achieve effective cross-cultural communication, whereas excessive security can lead to ethnocentrism and insufficient security can lead to fear of an unfamiliar culture. Ting-Toomey’s identity negotiation theory refers to the process of effectively managing and harmonizing one’s cultural identity during cross-cultural communication. It has been applied to identity management research based on participants’ cultural backgrounds in studies involving Assyrian women (Collie et al., 2010), Asian Caucasians (Dorjee et al., 2013; Toomey et al., 2013) and couples from different cultures (Martinez et al., 2016). According to Ting-Toomey (2007), mindfulness in dealing with cultural differences can be conceptualized as a multidimensional construct encompassing philosophical, spiritual, meditative, cognitive, affective, behavioral, and ethical components. She proposed four stages of intercultural competence: total mindlessness, semi-mindfulness, full mindfulness, and mindless mindfulness. The first three refer to the process of “self” development, which is “given” identity in intercultural contexts. Nevertheless, the last “mindless mindfulness” stage refers to the acquisition of a dynamic consciousness by individuals who can communicate in a highly adaptable manner across cultural contexts. By shifting between mindless and mindful consciousness, this cultural transformer demonstrates comfort through dichotomies, ambiguities, and uncertain intercultural circumstances.

AUM and identity negotiation theory provide different perspectives on identity in dynamic cultures. Toomey et al. (2013) proposed the model of “double-swing bicultural identity” and applied it to individuals’ integrative identities with a bi-cultural background capable of shifting from one culture to another depending upon the situational and communicative contexts (Toomey et al., 2013). During this process, an individual’s identity undergoes a series of negotiations, adaptations, and adjustments. Based on Gudykunst’s (1995, 1998) AUM Theory, Haiyan (2014) developed the Cultural Anxiety Questionnaire (CAQ). CAQ is the survey instrument employed by this research. It contains 12 items for evaluating the situations of anxiety in intercultural contexts, such as nervousness, refusal, discomfort, or apprehension. Haiyan’s (2014) study combines psychology, sociology, and intercultural communication theories to explore the cultural identity of Chinese EFL learners. However, both AUM and identity negotiation theory rarely examine the complexity of cultural identities closely.

In the intercultural context, no element, regardless of inter or outer context, should be considered alone. The eco-linguistic framework can provide a different perspective for analyzing the complexity of cultural identities. It presents the view that the development of human skills is affected by various factors, including “communities, action, perception, and languaging” (Steffensen and Cowley, 2021). The ecological approach takes a non-essentialist view and considers identity to be shaped by a complex open system with a multilayer micro-social timescale (Uryu et al., 2014). Multilevel approaches to ecological studies include macro-, meso-, exo-, and micro-level contextual factors. This framework can help researchers avoid the “reductionism to a single level that underestimates the effects of other contexts” (Oetzel et al., 2013). Some scholars (Ting-Toomey, 2010; Dorjee et al., 2013; Kilanowski, 2017; Dorjee and Ting-Toomey, 2020) have expanded the elements under study to include these four levels. Macro-level factors concern “the larger sociocultural contexts, histories, worldviews, beliefs, values, and ideologies”; meso-level factor analysis considers the impact of the family units, workplaces, or the local neighborhood; exo-level analysis pertains to the examination of formal institutions (e.g., government agency, school system) and mass media; and micro-level analysis encompasses both intrapersonal-level and interpersonal-level characteristics. To understand cultural identity construction, appropriately analyzing the complexity of the ecosystem is important. Multilevel approaches to the ecological framework are beneficial for research that copes with complexity (Oetzel et al., 2006; Oetzel et al., 2013).

Furthermore, the analysis of the eco-linguistic perspective examines ideologies, metaphors, frames, and other cognitive and linguistic phenomena to reveal the stories that affect people’s lives and society (Stibbe, 2021). As Stibbe (2021) states, “Narrative is the most powerful form of story…….” Narratives can be simple or complex and manifest in various forms, including oral storytelling, written works, and visual images. By analyzing the narratives of an unknown author who used the metaphor “boat journey” to describe COVID-19, Stibbe (2021) provides a specific research approach for eco-linguistics: the narration of unpredictable overwhelm incidents, such as COVID-19, is valuable for comprehending the connection between language and the ecosystem. Therefore, this research adopted NVivo to analyze the narrations and descriptions of Western and Chinese culture to explore the relationship between ecosystem and identity from the multi-scalar approach of eco-linguistics. In eco-linguistic research, NVivo is an effective tool for drawing categories, investigating trends, and developing theoretical models. Bryant (2017) argues that Computer Assisted Qualitative Data Analysis (CAQDAS) software may limit researchers’ thoughts and judgments. However, the drawback of NVivo can be reduced through a multilevel approach of eco-linguistics, which provides a holistic view of cultural diversity and broadens our research insights.

## 3. Materials and methods

This study comprises two studies, applying both qualitative and quantitative methods. In the qualitative part of both studies, the textual data are examined using NVivo, the qualitative part of Study 1 with NVivo 12.0, and that of Study 2 with NVivo 20.0. In the quantitative part, both Studies 1 and 2 adopt a questionnaire survey. Questionnaire 1 in Study 1 applies Haiyan’s (2014) CAQ. The initial copies of CAQ were distributed to 287 students to ensure credibility. The questionnaire dimensions were then slightly readjusted based on students’ language learning levels. The final version of Questionnaire 1 scored on a 5-point Likert scale with 12 items that include the attitudes toward the food, language, wedding style, festival, and opera in Western cultures. Besides, based on AUM theory and Ting-Toomey’s identity negotiation theory, Questionnaire 2 in Study 2 was an expanded version of Questionnaire 1 with 15 items. The three more items are about attitudes toward critical thinking, creativity, and the Western educational system, also scored on a 5-point Likert scale.

### 3.1. Samples

Studies 1 and 2 were carried out in this research at different times. Study 1 was conducted before the outbreak of COVID-19, while Study 2 was conducted during COVID-19. Eight hundred forty-two participants in this research were from two different groups of freshmen in one arts college. Before conducting the quantitative research in Study 1, students used to be asked to describe their feelings regarding Chinese and Western culture in English writing assignments, which was initially unrelated to this research. However, after accumulating essays, different and diverse attitudes toward Chinese and Western cultures emerged. Questionnaire 1 was distributed to the first group of 483 students and analyzed using SPSS 11.0. The assignments of students with specific anxiety and uncertainty levels were selected to be analyzed in the qualitative part of Study 1. In Study 2, another group of 359 students was required to write about their feelings about Chinese and Western cultures in an English writing course during the pandemic. Then Questionnaire 2 was distributed to select the participants for qualitative analysis in Study 2. As Stibbe (2021) states, the traditional story is a series of intentionally selected events, but the “story” in eco-linguistics unconsciously emerged in everyday life. Thus, the students were allowed to express their attitudes without length or extent limitations in assignments.

### 3.2. Research tools and data collection

#### 3.2.1. Reliability and validation of questionnaire 1

Study 1 was conducted before the outbreak of COVID-19. As shown in Table 1, Cronbach’s α for Questionnaire 1 is 0.871, demonstrating that the questionnaire is credible. Questionnaire 1 was distributed to 483 students and then analyzed using SPSS 11.0.

**Table 1:** The validation of questionnaire 1.

Reliability statistics		
Cronbach’s alpha	Cronbach’s alpha based on standardized items	N of items
0.871	0.873	12

#### 3.2.2. Reliability and validation of questionnaire 2

Study 2 was conducted during the COVID-19 pandemic. Table 2 displays that Cronbach’s *α* of Questionnaire 2 is 0.834, demonstrating the validity of the questionnaire. Questionnaire 2 was distributed to 359 students and then analyzed using SPSS 12.0.

**Table 2:** The validation of questionnaire 2.

Reliability statistics		
Cronbach’s alpha	Cronbach’s Alpha Based on Standardized Items	N of Items
0.834	0.840	15

### 3.3. Content analysis

Once the quantitative analysis of each study was finished, the content analysis was conducted. Text versions of the data in both qualitative analyses of Studies 1 and 2 were generated and then imported into NVivo. Study 1 was conducted before the latest version NVivo. Therefore, Study 1 employed NVivo 12.0, while Study 2 used the latest version, NVivo 20.0, for analysis. The results of Studies 1 and 2 differ in the type of figures produced by NVivo. In NVivo 12.0, the data analysis is challenging to visualize, but in NVivo 20.0, the relationship between elements and categories can be easily illustrated by the figure. This feature promotes a more efficient data analysis, allowing for a more comprehensive understanding of complex data sets.

## 4. Result

### 4.1. Study 1

#### 4.1.1. The selection of participants according to AUM theory

**Figure 1:**
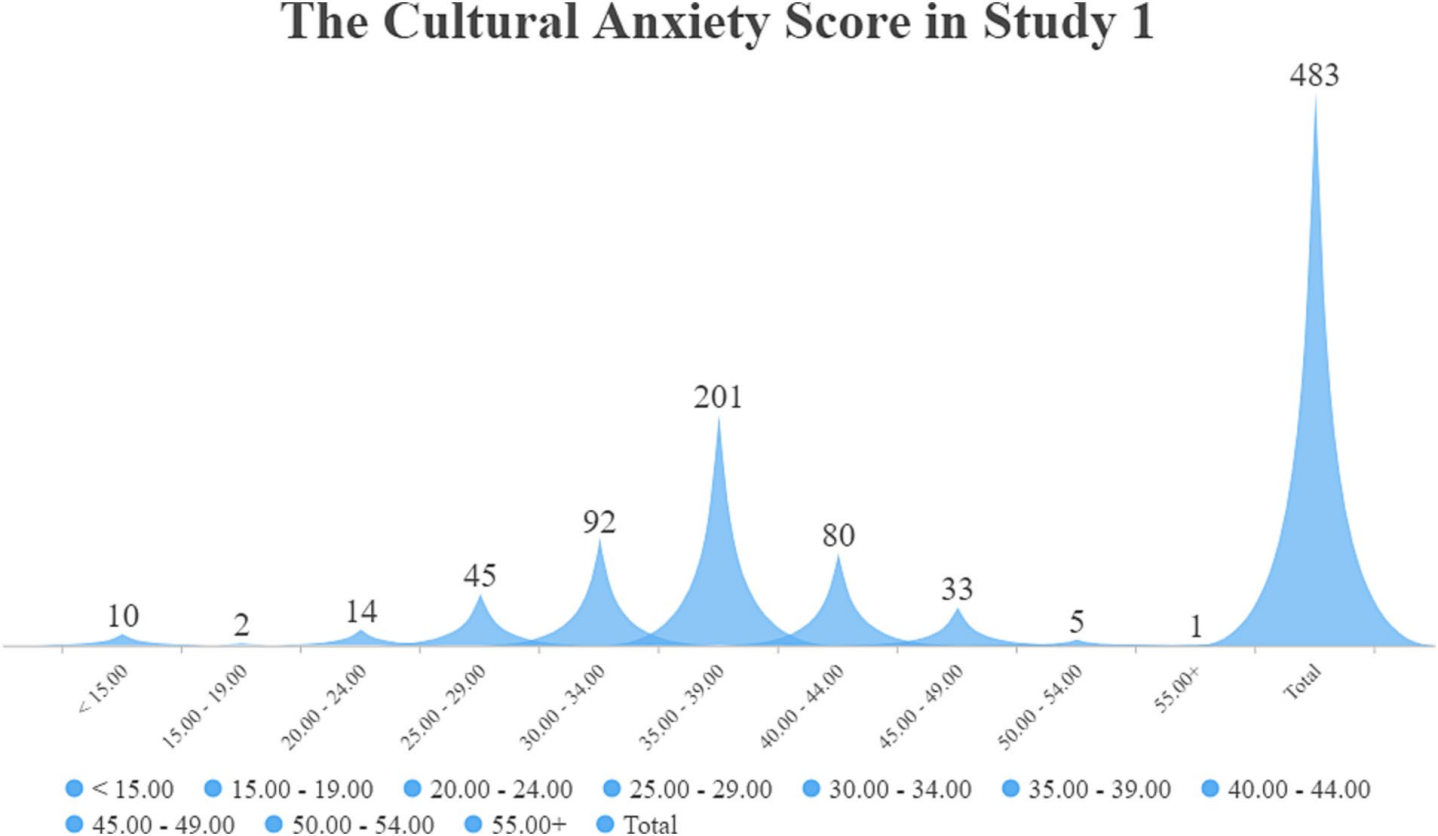
The numbers of participants in every score section of Study 1.

The total score on Questionnaire 1 was 60. According to Gudykunst (1998) and Matveev (2017), individuals with cultural anxiety and uncertainty between maximum and minimum levels should be competent in intercultural communication. Therefore, in Study 1, participants who obtained scores between 30 and 34 are supposed to be at the ideal cultural anxiety level. Table 3 demonstrates that the mean of the cultural anxiety level is 35.69, which is higher than the ideal level (30–34) of cultural uncertainty and anxiety. Figure 1 shows that a total of 92 participants are in this level(30–34), and 20 writing assignments of these 92 are chosen randomly to be analyzed in NVivo 12.0.

**Table 3:** The average score of the participants in Study 1.

Scale statistics			
Mean	Variance	Std. deviation	N of items
35.69	46.512	6.82	12

#### 4.1.2. The analysis of cultural identities of Chinese EFL learners before COVID-19

##### 4.1.2.1. Cultural identity construction

In order to explore the cultural identity of the EFL learners in the ideal level of cultural uncertainty and anxiety, the interview questions cover as much as possible aspects of Chinese and Western cultures. The data were transcribed in texts and analyzed using NVivo 11.0 (S1, S2, S3….S20 in the following subsections represent the participants.)

**Figure 2:**
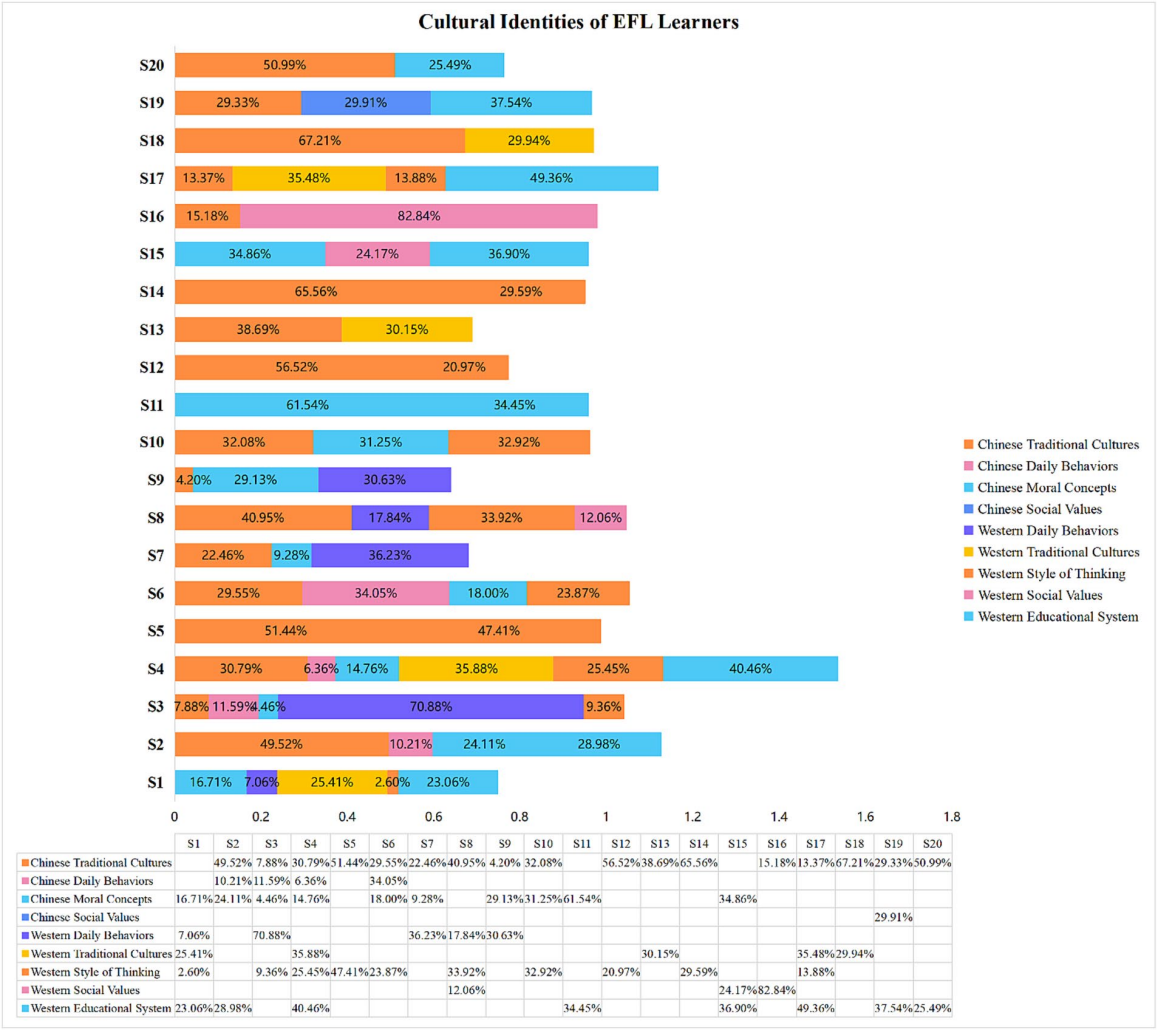
Cultural identities construction of EFL learners.

As shown in Figure 2, four major categories were organized for Chinese cultural identities: Chinese social values, Chinese traditional cultures, Chinese moral concepts, and Chinese s daily behaviors. Five major categories were then organized for Western cultural identities: Western daily behaviors, Western traditional cultures, Western style of thinking, Western social values, and Western educational system.

In general, 11 participants (S2, S5, S6, S10, S11, S12, S13, S14, S18, S19, S20) identified more with Chinese cultural identities. Chinese traditional cultures accounted for a significant proportion of the four categories of Chinese identity. Seventeen participants mentioned the content of Chinese traditional culture to various degrees, with five participants covering more than 50%: S5 (51.44%), S12 (56.52%), S14 (65.56%), S18 (67.21%), and S20 (50.99%). Chinese moral concepts were also an important category, and 10 participants (S1, S2, S3, S4, S6, S7, S9, S10, S11, S15) described the content of moral concepts in their daily lives. Additionally, compared with Chinese traditional cultures, Chinese social values formed the smallest portion of the four categories. The moral concepts were not combined with social values because the research findings showed that most participants identified Chinese social values at a micro-analytical level, which focuses on the relationship between participants and their family members.

*S11: Chinese culture attaches great importance to family and friends and regards them as the cells that make up society. However, in Western countries, independence and self-reliance are advocated. Regarding family affection, the Chinese have more affection than the Westerners.*

*S15: I identify with Chinese culture in terms of filial piety. The promotion of filial piety is part of the long history of Chinese culture, a traditional culture that has continued from ancient times to the present day. From childhood to adulthood, the Chinese may have lived with their parents for a long time, while Western children grow up more independently.*

All of the participants’ cultural identities were neither fixed nor immutable. Six participants (S1, S3, S4, S8, S15, and S16) identified characteristics that were more Western than Chinese and maintained a balance between Western and Chinese cultural identities, preferring a comparatively open Western cultural environment while also identifying with traditional Chinese culture. In addition, Western daily behaviors and social values were two categories that participants S3 (70.88%) and S16 (82.84%) demonstrated at high levels.

S3 identified the Western daily behaviors specifically and also approved of the traditional Chinese moral concepts:

*S3: Western culture pays more attention to the privacy of others in daily communication; it is a kind of respect for each other. Westerners do not disclose the privacy of their lives when communicating, and they are not interested in other people’s privacy. I also prefer the emotional communication of the Western style. In Western culture, parents and children, brothers and sisters, and friends always feel free to show their emotions to each other. They will use body language like hugging and kissing to express their feeling. Chinese culture lacks body language to express feelings and share between people, but I still identify the Chinese moral concepts. Chinese culture puts filial piety first, paying more attention to the moral value and seniority in the family or clan.*

Despite having the highest coverage ratio for Western cultures and values, S16 did not fully approve of Western culture, but she approved of Confucianism.

*S16: One of the values that resonates with me from Confucianism is benevolence and love for others, which is encapsulated in the golden rule, “Do not do unto others what you would not have them do unto you.” However, I personally prefer Western cultures. In contrast to Chinese culture where disobedience toward parents is considered a sign of lack of filial piety, Western parents provide guidance while allowing their children to make their own choices. For example, Western parents will not care about whether you want to get married at the age of 18 or 70 or not to get married at all, because this is your own business. Western parents do not use the concept of filial piety to manipulate their children.*

Moreover, Figure 2 shows that the participants’ cultural identity is not wholly influenced by any single culture; instead, they can adopt an inclusive but rational attitude toward cultural differences. Three participants (S7, S9, and S17) belonged to Chinese and Western cultures. S7 and S9 identified Chinese traditional cultures and moral concepts but commented positively on Western daily behaviors.

*S7: I respect both Chinese and Western cultures. Each region has unique cultural customs, and it is not appropriate to judge any country’s culture. For instance, Westerners prefer to shower in the morning to start their day feeling clean and refreshed, while the Chinese prefer to take a bath at night. Personally, I prefer the Western way. Additionally, Westerners tend to be more outgoing and enthusiastic when meeting others, while the Chinese tend to be more reserved. In this regard, I personally prefer the Chinese way of communication.*

*S9: Both Chinese and Western cultures have rich and diverse histories. While there are significant differences between them, it’s not accurate to say that one culture is superior to the other. In fact, the unique aspects of both cultures contribute to the richness of global culture. Personally, I appreciate the value Western culture places on privacy and the adventurous spirit of its people. Unlike in China, Westerners tend to avoid asking personal questions in public. Additionally, the Western spirit of exploration and innovation is truly inspiring. However, I also deeply value the strong family ties in Chinese culture. The Lunar New Year is a time when families come together, no matter how far apart they may be.*

S17 commented positively on both Chinese and Western traditional cultures. She also praised Western styles of thinking and educational systems.

*S17: Chinese and Western cultures are different. Western culture tends to be more flamboyant and unrestrained, while Chinese culture values restraint, observance, thrift, and simplicity. I prefer Western culture and have become more interested in it through studying English. I appreciate the exploratory and discovery-based teaching methods in Western education, but I also believe in the importance of Chinese etiquette and customs. Respect for elders and filial piety has been integral to Chinese culture since ancient times, and these values should not be abandoned.*

##### 4.1.2.2. Contextual level factors affecting cultural identity

Because culture should not be viewed as an unchanging concept and cultural identity should not be viewed as isolated and static, it is significant to recognize the complex differences in the factors that influence an individual’s cultural identity. In the socioecological framework, the diversity of individual cultural identities can be understood through macro-, exo-, meso-, and micro-level analyses. Dorjee and Ting-Toomey (2020) stated that elucidating the characteristics of cultural identity and its influencing factors at multiple levels of socio-ecological perspectives is an effective method for comprehending the situation of cultural identity in an intercultural context.

**Figure 3:**
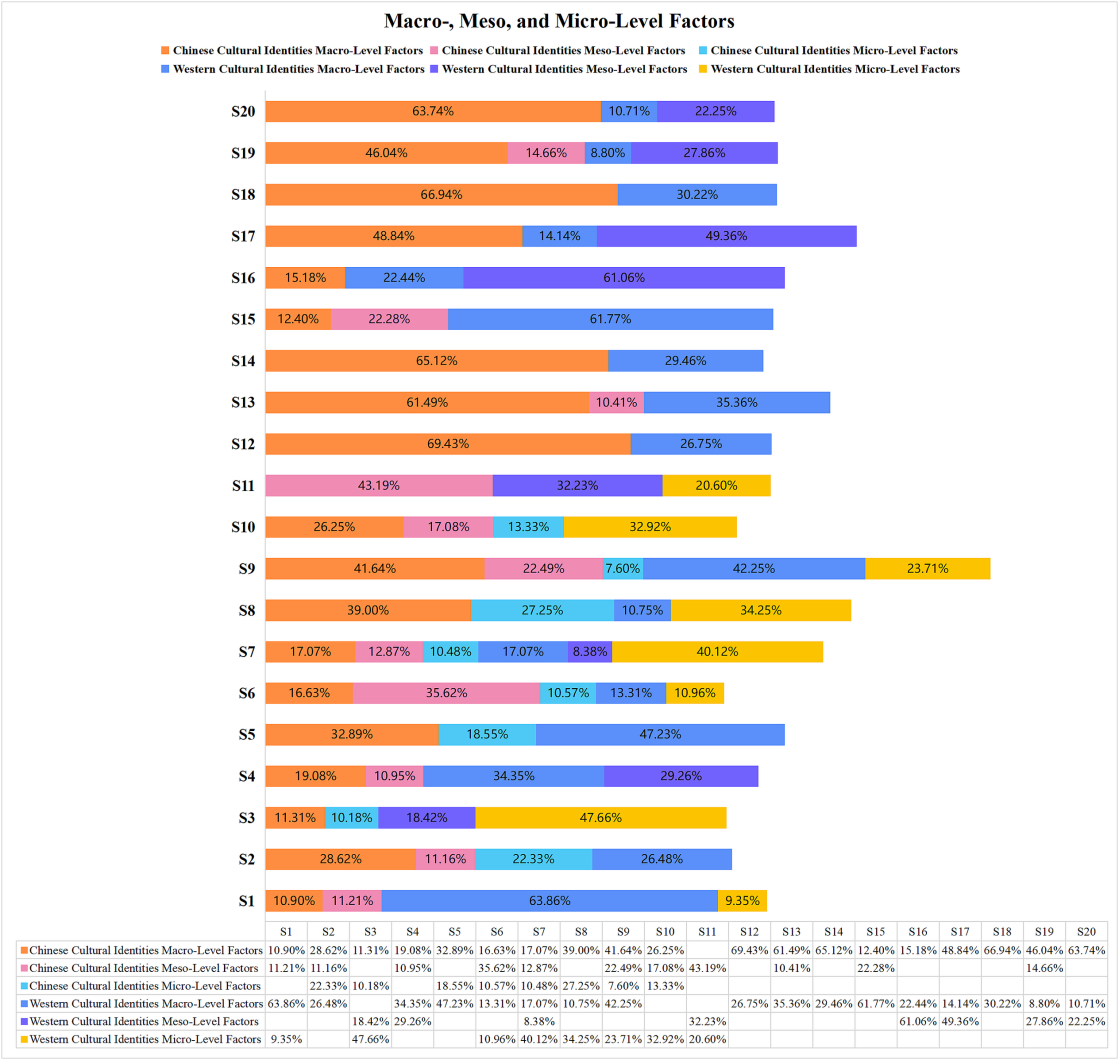
Contextual level factors.

As demonstrated in Figure 3, the multiple-level factors influencing cultural identities were classified into macro-, meso-, and micro-levels. However, the exo-level factor was not included. First, macro-level factors, including sociocultural contexts, histories, worldviews, beliefs, values, and ideologies (Dorjee and Ting-Toomey, 2020), have a significant impact on the cultural identities of the majority of participants. In the result of Study 1, macro-level factors include Chinese traditional cultures and values, traditional Western culture, and Western social values. Figure 3 shows that the cultural identities of S3 and S10 were affected only by macro-level factors in the Chinese context. In contrast, the cultural identities of S11 were only affected by macro-level factors in Western circumstances. In addition to these three participants (S3, S10, S11), the remaining seventeen’s cultural identities were affected by macro-level factors in both the Chinese and Western contexts. Among these 17 participants, there were five (S12, S13, S14, S18, S20) whose cultural identities were affected by macro-level elements of Chinese circumstances by more than 60%, but two (S1, S16) were affected by macro-level elements of Western circumstances by more than 60%.

Meso-level factors demonstrate the influence of groups, communities, and organizations can significantly affect the cultural identity. In Study 1, the meso-level include Chinese moral concepts, Western daily behaviors, and the Western educational system. Figure 3 illustrates that at this level, five participants (S5, S8, S12, S14, S18) never appeared at the meso-level in either Chinese or Western circumstances. Seven participants (S1, S2, S6, S9, S10, S13, S15)were exclusively influenced by the meso-level factors of Chinese circumstances, and S6 had the highest coverage ratio of Chinese meso-level factors(35.62%). By contrast, four participants(S3, S16, S17, S20) were only affected by the meso-level factors of Western circumstances, and S16’s cultural identity had the highest coverage ratio of Western Meso-level factors(61.06%). Furthermore, the cultural identities of the four participants (S4, S7, S11, S19) were affected by Chinese and Western meso-level factors. According to Dorjee and Ting-Toomey (2020), the meso-level factors are typically associated with family relationships. This finding indicates that these four participants not only appreciate traditional Chinese family concepts, but also desire a relatively autonomous and independent parent–child relationship in Western culture.

Micro-level analysis encompasses both intrapersonal- and interpersonal-level characteristics (Dorjee and Ting-Toomey, 2020). Intrapersonal-level features represent the construction and negotiation of cultural identity, whereas interpersonal-level features represent how individuals in a particular culture carry out their daily activities and deal with conflicts. In this study, the micro-level factors included Chinese daily behaviors, Western daily behaviors, and Western thinking styles. Micro-level analyses of cultural identities tend to be more individualized and focus on the characteristics of emotions and personality in Chinese and Western cultures. As demonstrated in Figure 3, compared with the macro- and meso-levels, the micro-level was the smallest. The cultural identities of the ten participants (S4, S12, S13, S14, S15, S16, S17, S18, S19, S20) did not involve micro-level factors. Notably, only S2 and S5 were exclusively affected by the micro-level elements of Chinese cultural circumstances, whereas Western cultural circumstances influenced S1 and S11. Furthermore, six participants’ cultural identities (S3, S6, S7, S8, S9, S10) were affected by micro-level factors in both Chinese and Western cultures. Specifically, these six individuals assimilated the emotional nuances of Chinese culture, such as modesty, subtlety, and restraint, while also striving to retain their unique personal identities during social interactions in Western settings. This finding highlights the complex interplay between cultural identity and social context and underscores the importance of sensitivity and awareness when navigating cross-cultural interactions.

### 4.2. Study 2

#### 4.2.1. Selection of participants according to AUM theory and identity negotiation theory

**Figure 4:**
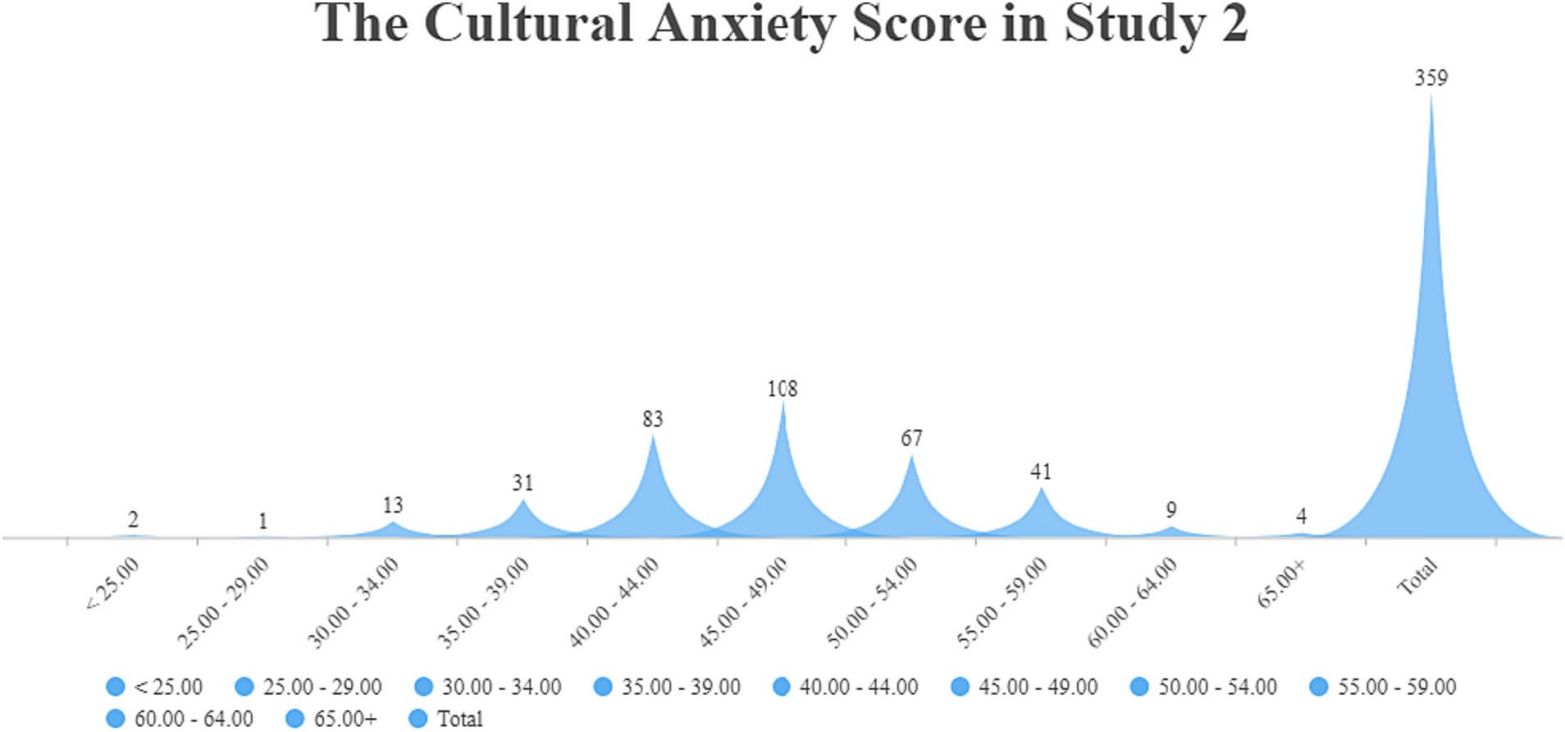
The numbers of participants in every score section of Study 2.

The total score on Questionnaire 2 was 75. As shown in Table 4, the *mean* of the cultural anxiety level is 46.94, which was higher than the ideal level (35–39) of cultural uncertainty and anxiety. Generally, the research findings for Questionnaires 1 and 2 showed that the participants had high cultural uncertainty and anxiety. In the result of Questionnaire 1, Figure 1 demonstrates that 201 participants are in the high cultural anxiety level of 35–39, which accounts for the largest part of 483 participants. In the result of Questionnaire 2, Figure 4 demonstrates that 108 participants are in the high cultural anxiety level of 45–49, which still accounts for the largest part of 359 participants. In the English writing assignment, the participants are required to answer several specific questions about Chinese and Western cultures, demonstrate their opinion about the two cultures pre- and post-COVID-19, and then write an essay based on their viewpoints. As seen in Figure 4, only 31 participants are in the ideal cultural anxiety level (35–39). Twenty assignments of these 31 participants are selected randomly for qualitative analysis. The data of their view about Western and Chinese cultures in their assignment are analyzed in NVivo 20.0.

**Table 4:** The average score of the participants in Study 2.

Scale statistics			
Mean	Variance	Std. deviation	N of items
46.94	54.47	7.38	15

#### 4.2.2. The construction of cultural identities after the COVID-19 pandemic from an eco-linguistics perspective

Language identity is multidimensional and encompasses various elements in a dynamic cultural context containing many aspects. From the perspective of eco-linguistics, language is part of the ecosystem. The cultural identity analysis lens provides an angle to examine language and culture individually. It addresses the connections among macro-, meso-, exo-, and micro-level factors in an intercultural context.

##### 4.2.2.1. Macro-level factors

**Figure 5:**
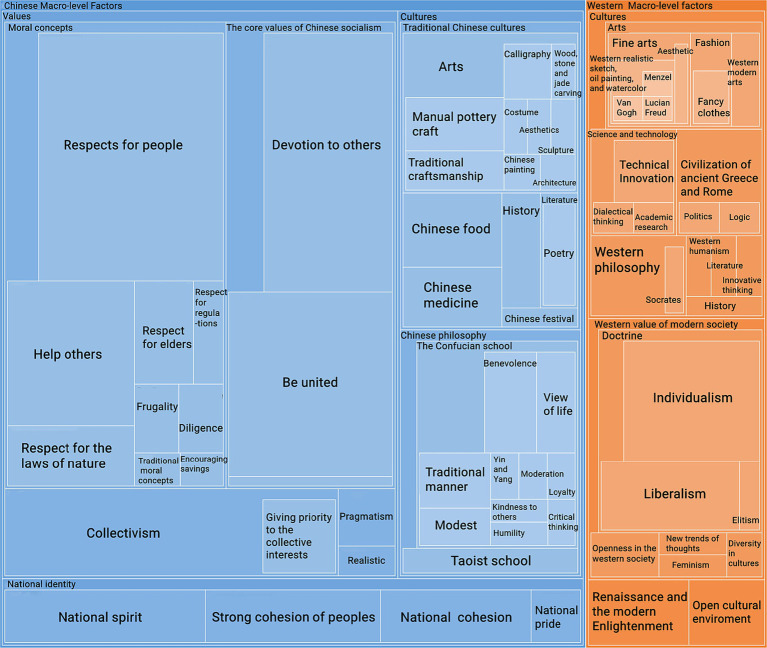
Macro-level factors in both Chinese and Western cultures.

As demonstrated in Figure 5, the macro-level factors in Chinese and Western circumstances are the most significant part of the participants’ cultural identity. This is also revealed by the research findings of Study 1 (Figure 3). Western macro-level factors can be separated into two parts: Western value of modern society and Cultures, and Chinese macro-level factors can be separated into three parts: Cultures, Values, and National identity. National identity can be defined as the conceptualization of nationality based on portraying a national community founded on a perceived common lineage (Pehrson et al., 2009). In the participants’ narration, national identity was intertwined with culture. Macro-level factors in both Chinese and Western circumstances encompass many elements of culture and values. Before COVID-19, the participants rarely mentioned their national identity during the interviews, and the most impressive macro-level factors in the interview data were traditional cultures and moral concepts. During this post-epidemic period, the research findings show that they acknowledge their national identity more.

*S10: The strong sense of unity and cohesion embedded in Chinese culture has been instrumental in overcoming the challenges of COVID-19. We worked together to achieve remarkable success in controlling the epidemic in China, driven by the motivation of the entire nation. The progress made in the fight against the virus has been truly inspiring.*

*S17: The spirit of unity in Chinese culture is something I agree with very much, such as fighting together and dedication during COVID-19. In Chinese culture, every word carries weight. We promise, then we commit. These also make us more united and share responsibility during COVID-19. These national spirits support us in overcoming the hardship of the epidemic.*

Moreover, traditional Chinese culture is a significant macro-level factor that substantially impacts participants’ identities. Fine arts play a crucial role in culture, but they are seldom included in the literature review of cultural identity, let alone in studies on social-ecological systems. The art elements in the macro-level factors mostly remained the same before and after COVID-19, which can be considered the most stable part of cultural identity. Dorjee et al. (2013) and Dorjee and Ting-Toomey (2020) applied the social-ecological view to analyze cultural conflict but did not mention the role of art in the connection between cultures. Most Chinese and Western macro-level cultural elements are related to the arts. In Chinese culture, calligraphy, costume, aesthetics, wood, stone, and jade carving, traditional craftsmanship, manual pottery craft, and Chinese sculpture, painting and architecture are included in the participants’ descriptions. In Western cultures, most factors concentrate on modern art aesthetics and fashion, while others focus on traditional Western realistic sketches, oil paintings, and watercolors. It is worth noting that the aesthetic perspectives in both Chinese and Western cultures are mentioned in the description.

Furthermore, values are essential to both the Chinese and Western macro-level factors. From the perspective of resolving the pandemic problems, individualism and liberalism receive relatively negative remarks among participants in the values of contemporary Western society. However, when it comes to pursuing individual success in modern society, Western values receive many positive comments. Both feminism and elitism at the Western macro-level receive positive comments from participants. Besides, Chinese values contain moral concepts, modern values, and collectivism. Compared to the arts, which are the most stable elements in macro-level factors, moral concepts changed considerably during the pandemic period. After the onset of the COVID-19 pandemic, Chinese moral concepts primarily emphasize the public’s spirit and fortitude to face the challenges of the disease. Usually, the moral concepts in Chinese cultures are more about the personality in the family, such as diligence, frugality, and devotion to family, and therefore the Chinese moral concepts are categorized in Meso-level factors before COVID-19. However, influenced by COVID-19, the moral concepts in Chinese cultures are mainly focused on the relationship between the individual, the public, and the nation. Traditional moral concepts like diligence, frugality, and pragmatism are reflected in how individuals deal with the crisis of COVID-19. At the same time, the moral concepts encompassed during the pandemic period are the modern values in Chinese cultures, such as respecting others, respecting regulations and laws of nature, or devotion to others.

##### 4.2.2.2. Meso-level factors

**Figure 6:**
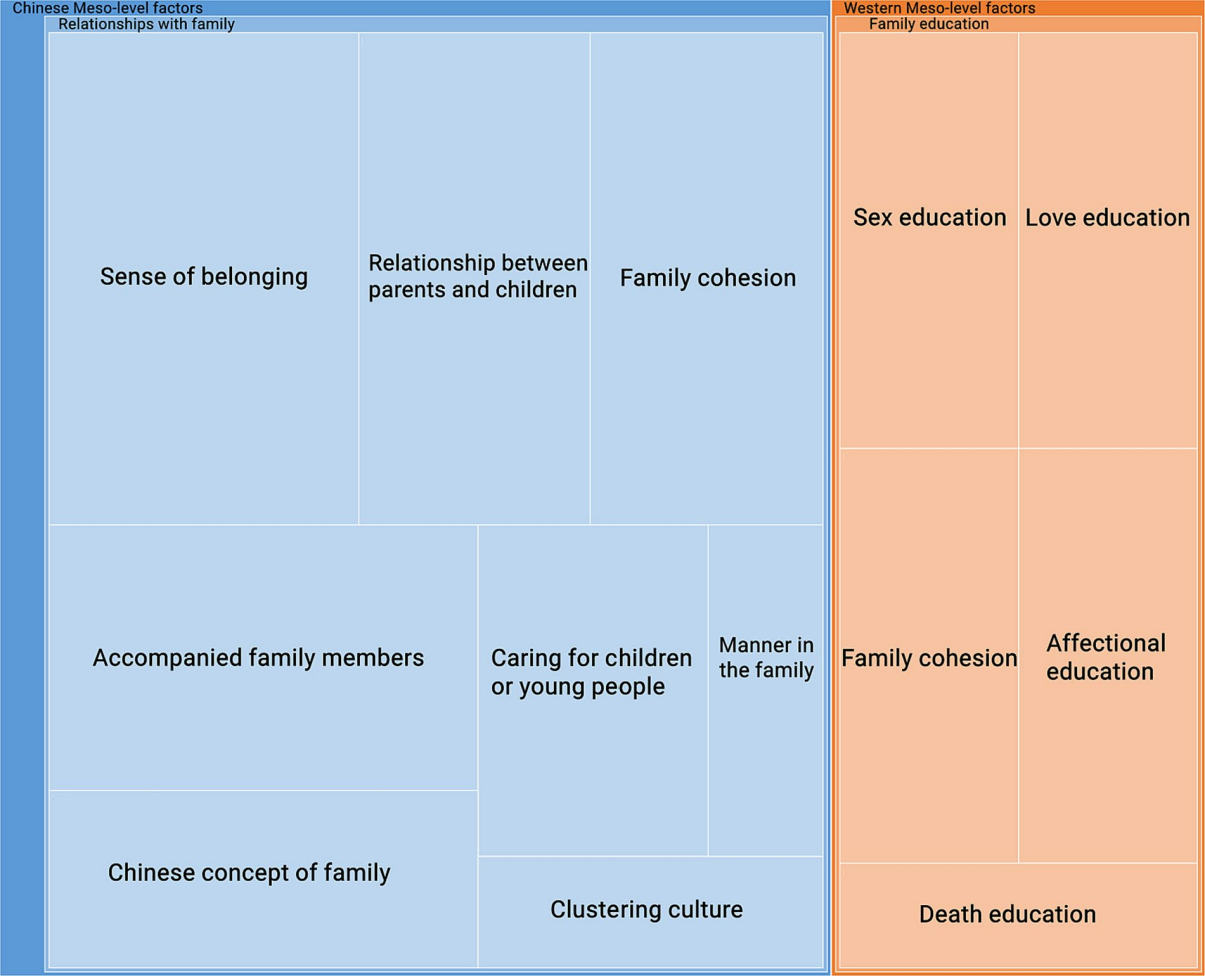
The meso-level factors.

Compared with the macro-level factors, there were relatively fewer meso-level factors. Figure 6 shows that the Chinese meso-level factors mainly focus on family relationships, while the Western meso-level factors focus on family education. In the results of Study 1, Figure 3 shows that the family data also concentrated on moral concepts, which have been categorized into meso-level factors. In the Chinese meso-level factors of Study 2, relationships with family can be divided into a sense of belonging, relationship between parents and children, family cohesion, accompanying family members, caring for children or young people, manners in the family, Chinese concept of family, and clustering culture. Compared to the data before COVID-19, attitudes toward family concepts have switched from morality to the feeling of getting along with family members in Chinese culture. This finding indicates that participants valued the time spent with family members after the pandemic.

*S10: In Chinese culture, parents traditionally take care of their children from childhood to adulthood, and the Lunar New Year is a time for families to come together, creating a strong sense of family unity. This is in contrast to Western culture, where the bond between adult children and their parents may weaken over time, leading to a gradual decline in family closeness.*

*S2: After COVID-19, the epidemic has dramatically affected many people’s family lives, and “home” has become essential. However, the relationship between the family and society has undergone subtle changes during the epidemic. Staying at home gives people more time to spend with their families and gives them more leisure time to learn traditional Chinese culture. We have gained a unique understanding of traditional culture, a sense of belonging, and identity in Chinese traditional cultures as we spend more time with our family.*

Moreover, the Western meso-level factors primarily focused on family education in Study 2, which was not included before COVID-19 in Study 1. The family education in Western meso-level factors includes sex education, love education, affectional education, death education, and family cohesion. The participants mainly appreciated the way they got along with their families in Western cultures in both Studies 1 and 2. The difference between family descriptions in Western cultures before and after COVID-19 lies in the concept of family cohesion. The participants in Study 1 made negative comments about reduced family cohesion in Western cultures. However, in Study 2, the participants thought that children should be raised to be independent, as a way to build their confidence.

*S13: I identify with Western culture in family education, love education, sex education, death education, and attitude toward animals. Western family education plays a significant role in children’s growth, encouraging children to be more independent.*

*S14: Independence holds great importance in Western culture. I came across a video of a blogger traveling in Denmark who met three children selling homemade drinks to fund their trip to Switzerland the following year. This is a commendable approach toward achieving their short-term goals through personal efforts, rather than depending solely on their parents, as encouraged in Western culture.*

*S16: I identified more with China’s cluster culture before the pandemic. In Chinese culture, people prefer bustle and gathering. However, Western culture tends to prioritize independence, with many people preferring to be alone. After the epidemic, I will identify with this independence of Western cultures, which is more conducive to preventing the spread of the virus during COVID-19.*

##### 4.2.2.3. Exo-level factors

**Figure 7:**
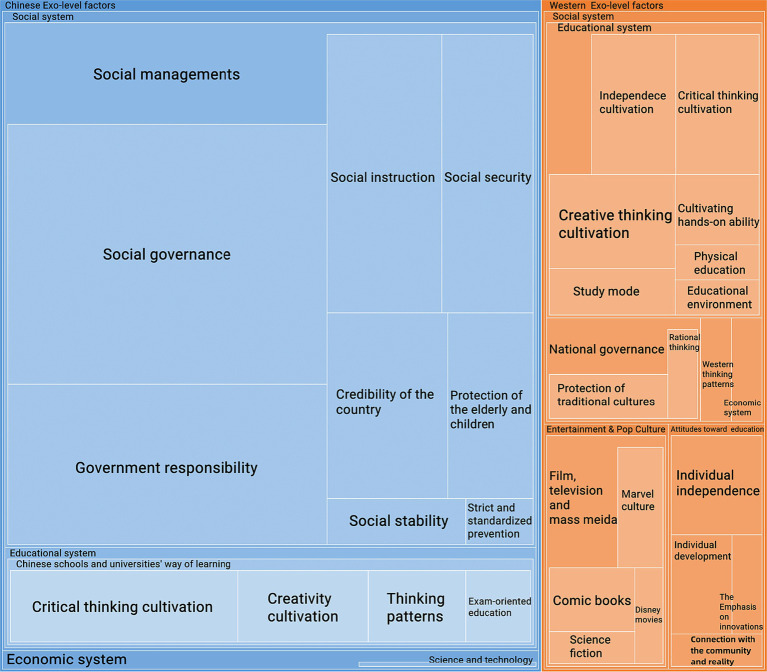
The exo-level factors.

Figure 7 illustrates the exo-level factors that emerged in this study. Newly identified exo-level factors are revealed in the findings of Study 2. The concept of exo-level analysis pertains to the examination of formal institutions, such as government agencies, courtroom, police, religious, healthcare, and school systems, which possess power resources and established personnel with defined roles and responsibilities (Dorjee and Ting-Toomey, 2020). According to Dorjee and Ting-Toomey (2020), digital and mass media factors can be categorized into exo- or meso-inquiry levels. Before COVID-19, there were fewer data for exo-level factors than for macro-, meso-, and micro-level factors. After COVID-19, attitudes toward Chinese and Western educational systems have mainly focused on ability cultivation. This finding indicates that the participants considered schools and universities to be the most influential institutions in terms of their academic ability.

Chinese exo-level factors include three categories: Social system, Educational system, and Economic system. Social systems can be divided into social management, social governance, social instruction, social security, social stability, government responsibility, the credibility of the country, protection of the elder and children, and strict and standardized prevention. The educational system is about the attitudes toward Chinese schools and universities, which can be divided into critical thinking and creativity cultivation, thinking patterns, and exam-oriented education. The economic system was the least exo-level factor and contained only one element: science and technology.

As mentioned above, there are few data on the Chinese education system in the findings of Study 1. However, Study 2 showed that participants had negative views toward the system, especially regarding creativity and critical thinking, as shown in Figure 7.

*S11: Our education prioritizes efficiency more than everything. After years of college study, many students’ thinking patterns become rigid, and they may lack critical thinking skills.*

*S8: Chinese students are trapped in the education model, and their ability to make innovation may be weaker. Most students only can think independently in university. An experiment showed that Chinese college students were better than Western students in their first year, but their critical thinking skills cannot be compared to Western students in their junior year.*

Social management in the exo-level factors of Chinese culture was also newly identified in Study 2. This refers to how people feel about the way institutions behaved during the COVID-19 pandemic. Participants in the study were impressed by how institutions in China handled the situation, including their methods of measurement, instruction, and responsibility.

*S5: Despite the severity of the epidemic, the Chinese people have displayed remarkable cooperation and confidence. In a very short period after the outbreak of the epidemic, we take corresponding measures to control the epidemic relatively quickly and effectively.*

*S17: My cultural identity is deeply rooted in various aspects of Chinese society, such as its social, traditional, and educational systems. In times of crisis, the Chinese people come together to propose solutions and provide support to solve problems. This solid system has proven resilient even in the challenges of COVID-19.*

Western exo-level factors comprise two categories: Social systems and popular cultures. The social system is primarily concerned with the educational system and national governance. Educational systems were found before and after the COVID-19 pandemic. In Study 1, Figure 2 shows that most participants made positive comments about the Western educational system, especially its learning and teaching methods, but nothing about the Chinese educational system. As most of these remarks are specific and limited to classroom experience, comments on the Western educational system are categorized as meso-level factors in Study 1. Moreover, Figure 7 shows that independence, critical thinking, and creativity cultivation were the significant exo-level factors in Western cultures.

In the participants’ opinions, Chinese students have experienced an education model characterized by strictness, leaving little room for critical and creative thinking. In addition, society has placed a strong emphasis on academic achievement, sometimes even idolizing it. They also believe that the Western educational system has had a positive impact on providing students with a greater chance to think critically and creatively. As seen in Figure 7, the participants’ attitudes toward Western education contain individual independence and development, emphasis on innovations, and connection with the community and reality.

*S11: The education in many Western countries pays more attention to inspiring and guiding critical thinking, providing more conditions for students to think creatively and speculatively.*

*S8: Western education is more inclined to educate students to think creatively, so Western students are generally more innovative and habitually think about the two sides of a thing.*

Additionally, entertainment and pop culture in relation to Western exo-level factors emerged after the COVID-19 pandemic. It mainly focuses on film, television, and mass media, such as science fiction, Disney movies, and comic books, especially the Marvel series. Participants believed that Marvel culture has emerged as a highly prevalent form of mass media in the contemporary society. Marvel creates many famous hero images, which has significant impact on Western popular cultures.

*S10: Hollywood is known for spending a significant amount of money on modifying the final product, as seen in the production of Avatar 2, which cost 350 million dollars. Most of the budget is allocated toward creating the artwork in Hollywood. However, it is interesting to note that the lead actors in our movies receive higher salaries than the team of Avatar 2.*

*S2: The Marvel culture, particularly the sight culture, is a dynamic and varied community of comic book figures brought to existence by a talented group of artists. This team comprises comic book writers, cartoonists, drama critics, filmmakers, and peripheral manufacturers who have collaboratively created a vast and intricate universe known as “The Avengers,” featuring many popular Marvel characters. They have breathed life into these characters by leveraging advanced comic book technology, developing a unique cross-industry cultural ecosystem.*

##### 4.2.2.4. Micro-level factors

**Figure 8:**
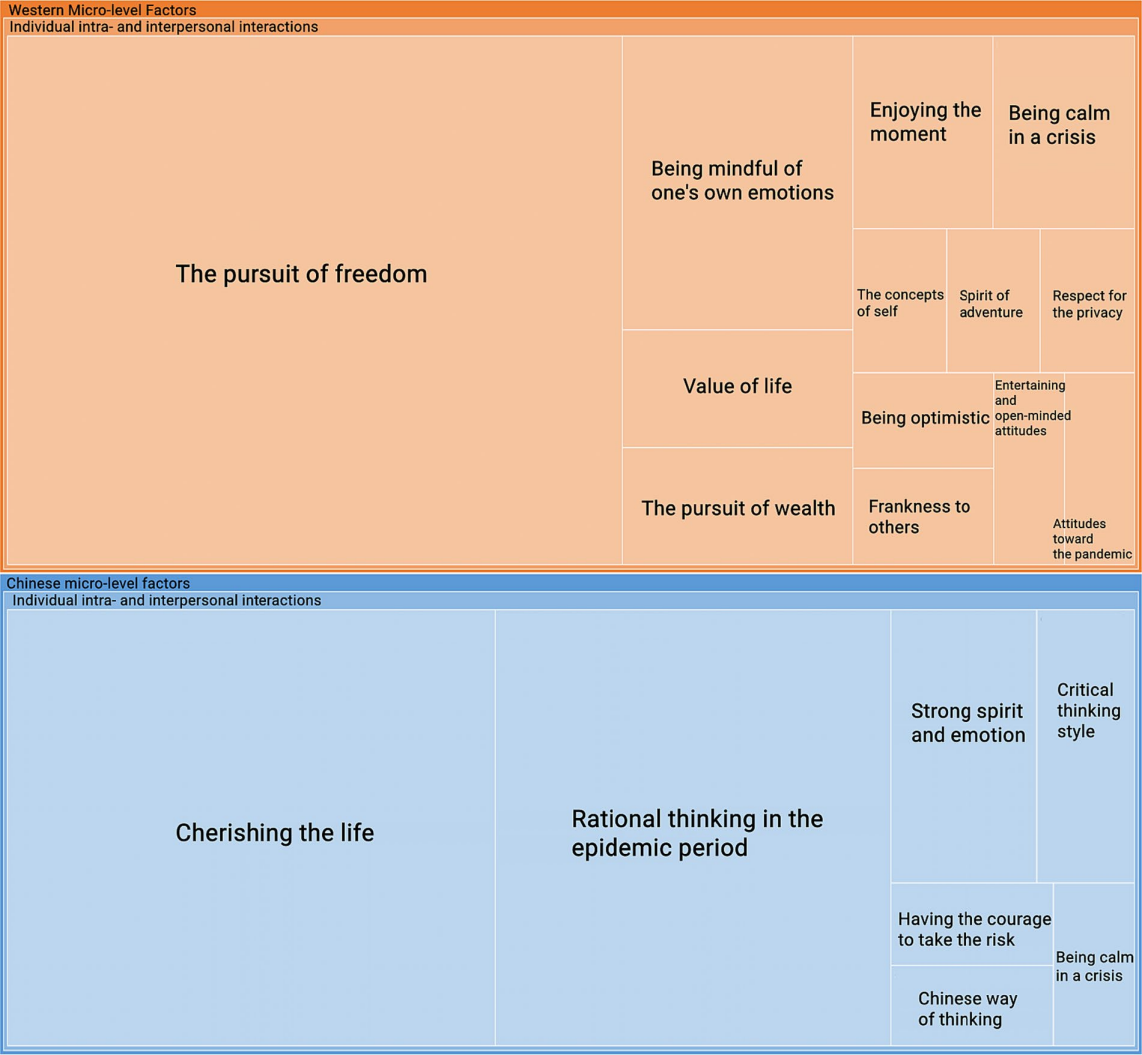
The micro-level factors.

The analysis presented in Figure 8 illustrates how different cultures approached conflict during the pandemic at a micro-level. In Study 1, Figure 3 shows that Chinese micro-level factors, such as modesty, humility, and introversion, were rooted in traditional Confucian values. However, in Study 2, these factors evolved significantly with communication methods deviating from conventional Confucian approaches. Figure 8 shows that the micro-level factors within Chinese culture do not encompass conventional Confucian intra- and interpersonal communication methods after COVID-19. By contrast, the Western micro-level factors in Study 1 were characterized by a focus on freedom and adventure. In the results of Study 2, these factors remained centered around the pursuit of freedom, a spirit of adventure, and respect for privacy, highlighting the differences in conflict resolution approaches across cultures. The findings of Studies 1 and 2 indicate that part of the micro-level components in Western cultures have remained remarkably stable both before and after the COVID-19 pandemic.

Figure 8 also demonstrates that the micro-level factors in Chinese culture changed during the COVID-19 pandemic. Individuals’ intra- and interpersonal interactions in Chinese micro-level factors included a strong spirit, thinking rationally and critically during the epidemic, courage to take risks, and being calm in a crisis. Compared with the research findings of Study 1, none of these elements appeared before COVID-19. According to Dervin (2016), the presentation of individual behavior as defined and constrained by the cultures in which they live can result in essentialism. This implies that the view of culture can contribute to essentialism, which is a potential reason for stereotypes. In this research, COVID-19 is the incident that changes the stereotype about Chinese. Furthermore, it is worth noting that among these elements, “being calm in a crisis” also emerged in the Western micro-level factors, which indicates how individuals dealing with conflict can be diversified regardless of the culture.

*S10: In China, the principle of prioritizing life is highly valued. During the COVID-19 pandemic, the Chinese have consistently sought truth through factual data, while respecting science. They have continuously improved their prevention and control measures based on the evolving situation. Their strategies to combat the virus have proven to be both stable and adaptable. With a rational approach, the Chinese can offer objective and dependable solutions to help ensure stability during this epidemic.*

*S15: The Chinese think rationally and critically during the epidemic. They were aware of the danger posed by the epidemic and used information and resources available on the network to ensure their safety and well-being. This helped more people to obtain helpful information and take necessary precautions.*

Besides, Figure 8 shows that the individuals’ intra- and interpersonal interaction in Western micro-level factors contains the pursuit of freedom, being mindful of one’s own emotions, value of life, the pursuit of wealth, enjoying the moment, being calm in a crisis, the concepts of self, the spirit of adventure, respect for the privacy, being optimistic, frankness to others, entertaining and open-minded attitudes, and attitudes toward the pandemic. Among the above elements, except the factors about freedom, adventure, and privacy, the others are newly found data after COVID-19. The participants praised the individuals’ perspective on the pandemic in Western cultures.

*S1: The Westerners believe that death is inevitable and pay more attention to their spiritual world. They can enjoy the present moment more, and the fear of death is weaker than us.*

*S20: Westerners can still maintain an optimistic and indifferent attitude during the pandemic. For example, a bakery in the United States set up an epidemic-themed cake, putting masks and other elements on snacks, adding a small amount of fun to customers’ boring quarantine life. Amidst the pandemic, the world has become a frightening place. However, many Westerners are coping with an optimistic and open-minded attitude, which is influenced by their free and accepting cultural and social environment.*

Furthermore, in Study 2, some participants interpreted the concept of “the pursuit of freedom” in Western cultures differently, believing that Western cultures encourage open-mindedness and thus create a social environment that allows individuals to pursue their personal aspirations to the fullest extent.

*S15: Westerners have a strong determination to follow their own dreams and not be influenced by external factors. They value their inspiration and strive for personal desires. They are not constrained by their parents and are willing to work hard to achieve their goals. They also fearlessly pursue love and are not limited by others’ views.*

*S17: Western culture tends to embrace more openness in cultures, including the academic environment. It also tends to be more accepting of diverse cultures and tolerant of different groups. Western culture has played a significant role in human history by providing valuable elements that helped humans transition from the agricultural to the industrial age. However, it is important to note that Western culture still has limitations that can hinder human development. We should carefully consider and examine the advantages and disadvantages of any culture.*

## 5. Discussion and conclusion

Cultural identity is affected by one’s ecosystem, which includes both—one’s internal and external surroundings. According to Dervin (2016), the differences between “cultures” should not be exclusively ascribed to the different cultures themselves. He proposed that “diversity needs to become diversities” and stated that specific individuals should be respected for their interculturality rather than their original, limited, and pre-conceived cultural labels. Eco-linguistics provides a unique angle from which individuals’ cultural identities can be observed as emerging from the ecosystem instead of attributable to any particular surroundings (Kramsch and Vork Steffensen, 2008). Kramsch and Vork Steffensen (2008) stated that Norton’s view of identity has been criticized for being too structuralistic in language acquisition, and that an ecologically oriented approach to language education should relinquish the insistence on standardization within the realm of language instruction. Guided by the ecological research paradigm, our study delves into the impact of individual differences on cultural identity at the macro-, meso-, exo-, and micro-levels. As Finke (2017) stated, “All cultures are ecosystems of the mind […]. The cultural diversity of psychological ecosystem is the decisive orienting frame for all linguistics and even more of any kind of eco-linguistics.” The research findings of Studies 1 and 2 illustrate that the factors affecting cultural identity originated from participants’ attitudes toward their surroundings, which can be investigated from an objective, individualized, and specific perspective.

The multilayer approaches are proven to be effective for comprehending ecological perspectives. Macro-, meso-, exo- and micro-level factors of social situations have been explored by some scholars (Dorjee et al., 2013; Uryu et al., 2014; Dorjee and Ting-Toomey, 2020), but these four levels are seldom fully covered in these studies. As demonstrated in the research findings, Study 1 covers three levels of research: macro, meso, and micro; Study 2 covers all four levels. The results of Study 1 showed that an individual’s cultural identity was mainly shaped by macro-, meso-, and micro-level factors of Chinese and Western cultures in the period before the COVID-19 pandemic. In Study 1, most participants were involved in macro-level factors, but the elements differed depending on the culture. First, in Chinese macro-level factors, the elements primarily focused on traditional culture and virtues, ancient historical civilization, and other related facets. At the Western macro-level, these factors were characterized mainly by the Western education system, modern civilization, industrial products, and economic development. Second, meso-level factors focused primarily on the interaction between educators, students, and the student’s families. The participants’ cultural identifications for Chinese cultures were influenced by various factors originating from the traditional concepts of family and institution, including traditional relationship modes such as “filial piety” and “respecting teachers and valuing education.” Cultural identifications for Western cultures mainly focused on Western standards of parent–child and teacher-student relationships. Besides, the Western-style education mode played a significant role in shaping participants’ identities. Third, the micro-level factors that affected cultural identity can be attributed to the participants’ interpersonal communication with their relatives and friends. In Chinese culture, these factors are generally characterized by modest and reserved personality traits and implicit emotional expressions. Conversely, for the Western cultures part, the factors that affect participants’ cultural identity are primarily associated with personal lifestyle choices, such as promoting individuality, respecting privacy, and maintaining an independent self in interpersonal communication.

The eco-linguistic perspective for analyzing individuals’ surrounding contexts provides an angle for changing the static view of cultural identity. Study 2 shows that cultural identity construction after the COVID-19 pandemic was dramatically different from that before, as evidenced by the results from Study 1. Although traditional cultural elements are still the most influential macro-level factors, Chinese national identity was a new addition after the pandemic in Study 2. National identity has recently been examined as an isolated subject in some studies (Liu and Chang, 2021; Malhotra and Ramalingam, 2022; Salmon-Letelier, 2022; Wood, 2022; Komisarof et al., 2023; Li et al., 2023; Monscheuer, 2023; Rentschler et al., 2023; Zhang et al., 2023), though these studies mainly focused on the elements that shape national identity. However, in the research findings from Study 2, national identity was a macro-level factor that was strengthened after the COVID-19 pandemic. This indicates that the identification of a nation is not isolated and can be shifted by actual social incidents in the construction of a changeable cultural identity. Part of the Chinese national identity is usually grounded in the principles of Confucianism, which encompass collectivism and the pursuit of social harmony. Based on the findings of Study 2, the occurrence of COVID-19 reinforces some Confucian values, such as being united and socially responsible, respecting authority, and prioritizing the welfare of the community. For instance, most participants perceived the individual’s adherence to regulations as a viable resolution to confront this global crisis instead of as a restriction on freedom. Therefore, the appearance of national identity elements in the macro-level factors of Study 2 can be attributed to the unpredictable context of the pandemic.

The concept of holism is important in eco-linguistics, as it emphasizes the interconnectedness, interdependence, and interactional nature of language, while valuing diversity and adopting a descriptive approach (Kramsch and Vork Steffensen, 2008). As Kramsch and Vork Steffensen (2008) state, “eco-linguistic emphasis on contextuality and open systems that the researcher sees him/herself as participant, i.e., as related to the object system under investigation.” Thus, in the analysis of meso- level factors, a holistic approach is necessary when comparing Chinese and Western cultures. It is crucial to maintain a respectful and impartial stance while analyzing different cultures to understand their diversity and complexity. First, Chinese culture emphasizes filial piety and family obligations, while Western cultures prioritize individualism and personal goals. However, it is important to note that the attitudes toward the family concept of different cultures can vary within the broader sociocultural context. COVID-19 challenged our notion of the family and emphasized the significance of support within the family unit. The results of Study 1 show that the Western-style family relationship was more popular among the participants, indicating a preference for the free, relaxed, and independent family atmosphere in Western cultures. On the other hand, the findings of Study 2 show that the participants identified more with Chinese family concepts after COVID-19.

From an eco-linguistic perspective, studying the interplay between identity dynamics and social contexts provides insight into the impact of super-diversity on an individual’s motivation to acquire knowledge (Steffensen and Kramsch, 2017). This can be seen in the exo-level factors of the results in Study 2. The exo-level factors are newly found in Study 2 after COVID-19 and did not appear in the results of Study 1. During the pandemic, insights concerning critical thinking, creativity, and popular culture emerged as exo-level factors affecting the students’ cultural identity. First, due to COVID-19, ensuring accurate and reliable information has become even more crucial. To fight against the spread of misinformation during a pandemic, fact-checking and source verification have become more significant than ever. To some extent, the COVID-19 pandemic has motivated students to prioritize critical thinking. Second, COVID-19 created many challenges for college students. Although the pandemic has provided more leisure time for students, COVID-19 limited their resources for personal collaboration while increasing their reliance on technology. This shift may influence students’ attitudes toward creativity and their preferred mediums of expression. As demonstrated in the findings of Study 2, Western entertainment and pop culture emerged as notable exo-level factors during COVID-19. This has been particularly evident in the success and influence of Marvel films, which have enjoyed considerable attention and popularity during these challenging times. Through the eco-linguistic analysis, the exo-level factors reveal that the pandemic has a profound impact on individuals’ motivation for critical thinking, creativity, and cultural preferences.

Besides, eco-linguistics can contribute to understanding how language is used to discuss and address the crisis. This perspective allows a holistic analysis that considers various factors and dimensions of cultural identity. By analyzing the participants’ comments on social incidents, eco-linguistics provides a comprehensive approach to understanding the relationship between language, culture, and the environment. The impact of COVID-19 extends far beyond the realm of public health, significantly influencing our society on a global scale. This global pandemic has also inevitably highlighted the pre-existing inequalities and tensions within diverse cultures, bringing them to our attention and enhancing our reexamination of whether the conflicts are from cultural differences or stereotypes. This can be ascribed to micro-level factors, such as the sense of community and personal relationships, in the findings of this research. Based on the micro-level factors in the findings of Studies 1 and 2, it is evident that the participants’ interpretations of social incidents are varied. In Study 1, the micro-level factors made up the smallest part, mainly regarding daily behavior, such as “expressing emotions directly” in Western culture and “beating around the bush” in Chinese culture. Nonetheless, in Study 2, analysis of comments on behavior regarding COVID-19 yields new data which reveal that some typical Western traits, such as open-mindedness, optimism, and calmness, are highly appreciated by participants. Additionally, China exhibited remarkable fortitude and efficacy in navigating the crisis of the COVID-19 pandemic. In the findings of Study 2, Chinese citizens demonstrated exceptional personal attributes, including rationality, composure, and adaptability, that facilitate their handling of crises. The findings of Study 2 show that after the pandemic, the micro-level factors of cultural identity also changed. Throughout Studies 1 and 2, we embraced Stibbe’s (2021) view of dynamic cultural identity, which considers identity “an evolving story.” This indicates that understanding social incidents is also a process of building self-awareness. Thus, cultural identity is actively gained from the surrounding environment rather than constituting a passive cultural label.

In addition, language acquisition in identity construction can be conceptualized as the cognitive process of comprehending one’s sense of self-identity (Torop, 2014). EFL learners typically come from diverse cultural backgrounds. This research aims to examine the factors influencing EFL learning for non-native speakers in a globalized context. The samples were selected based on the results of the CAQ. All of them were supposed to be able to negotiate between the two cultures because their questionnaire scores were at the ideal range of cultural anxiety. The research findings reveal that an individual’s cultural identification is as flexible as the culture itself. Cultural identity is not isolated from any surroundings of individuals, even though the information from the internet, movie, or television, regardless of the accuracy of the information presented. Pidduck et al. (2022) selected participants from among employees with experience on international business trips and suggested that intercultural experience strengthens the multicultural identity that represents the ability to handle cultural differences. However, the participants of Studies 1 and 2 were freshmen from an arts college, and few had experienced learning abroad. Still, they had perspectives about Chinese and Western cultures.

Furthermore, motivation is a primary factor that affects second language acquisition (Norton, 1995; Darvin and Norton, 2015). However, Darvin and Norton (2021) further proposed that investment is not the ultimate purpose of motivation, and the “complexity of identity and the interplay of different desires shape learners’ investments in different situations.” According to Darvin and Norton (2021), identity can be viewed as “not only person-in-context but also history-in-person.” Based on this theory, each individual possesses distinct perspectives influenced by diverse elements that mold their comprehension of their environment, which certainly affects their language learning process. Torop (2014) expanded the boundaries of language to “primary modeling system” and “secondary modeling systems.” The secondary modeling system includes literatures, arts, and medias, which can function as meta-linguistic systems influencing an individual’s existence. These metalanguages help individuals recognize their connections to the world and construct distinct identities. Moreover, learners from Chinese cultural backgrounds may face unique challenges in EFL learning because of cultural differences. Chinese culture is characterized by a long history, traditional virtues, and a strong emphasis on family and teacher-student relationships. These factors shape Chinese learners’ cultural identities and impact their approaches to English learning. Traditional Chinese culture places a high value on education and the role of teachers, which can influence how Chinese learners view their EFL instructors. The perspective of eco-linguistics provides us with the angle to comprehend how individuals construct their cultural identity and struggle to comprehend cultural differences.

## 6. Limitations and future work

EFL learners usually face challenges created by cultural differences. Learners’ initial language and cultural background are additional variables that affect their acquisition of EFL. With these abundant and dynamic language system elements, this study explores the complexity of individuals’ cultural identities. The limitations of this study can be divided into two streams: the participants and the lack of longitudinal research. First, in the qualitative part of the research, to avoid the participants’ psychological bias, the participants were selected based on the AUM and identity negotiation theory, and they are supposed to communicate competently in an intercultural context. Future studies should explore other methods for selecting participants in eco-linguistics research by considering their language learning ability, intercultural communicative competence, and other scales in psychology and sociology. Secondly, Study 2 should have conducted longitudinal research among the same group of participants. However, since the participants in Study 1 had already graduated from college when conducting Study 2, Study 1 and Study 2 had to choose different groups of research participants. The longitudinal research on cultural identities would be a valuable contribution to future eco-linguistics research.

From the eco-linguistics perspective, the finding of this research illustrates that cultural identity can be affected by the multi-scalar factors from the surrounding context. Nevertheless, there is still plenty of space for future work. First, the finding of this research only demonstrates the traditional arts in macro-level factors in Chinese and Western cultures, but the arts can be various. Therefore, the position of arts in the ecosystem can be studied further. Secondly, based on the findings of Studies 1 and 2, EFL learners often associate their national identity with ethical and moral concepts embedded within their native language. Conversely, they tend to associate critical thinking skills with native English speakers. Future studies can focus more on the relationship between cultural identity, national identity, and critical thinking from the eco-linguistics perspective. As Darvin (2017) states, language “not only constructs meaning but also imposes power.” The eco-linguistics perspective presents a framework for exploring the ever-evolving cultural identities of individuals. It would be valuable to conduct further research on how to be “self” in such a dynamic language ecosystem.

## Data availability statement

The raw data supporting the conclusions of this article will be made available by the author, without undue reservation.

## Ethics statement

The studies involving humans were approved by Guangzhou Academy of Fine Arts ethics committee (KXLL2023001). The studies were conducted in accordance with the local legislation and institutional requirements. The participants provided their written informed consent to participate in this study.

## Author contributions

YP crafted the manuscript, planned, wrote, and completed it, related research was also expertly conducted by the same individual, demonstrating a comprehensive approach to the project.

## References

[ref1] Alexander R. Stibbe A. (2014). From the analysis of ecological discourse to the ecological analysis of discourse. Lang. Sci. 41, 104–110. doi: 10.1016/j.langsci.2013.08.011

[ref2] Bagiyan A. Y. Shiryaeva T. A. Tikhonova E. V. Mekeko N. M. (2021). The real value of words: how target language linguistic modelling of foreign language teaching content shapes students’ professional identity. Heliyon 7:e06581. doi: 10.1016/j.heliyon.2021.e06581, PMID: 33869833 PMC8035504

[ref3] Bryant A. (2017) Grounded theory and grounded theorizing: Pragmatism in research practice. New York, NY: Oxford University Press, 17–19

[ref4] Caldas S. J. Caron-Caldas S. (1999). Language immersion and cultural identity: conflicting influences and values. Lang. Cult. Curric. 12, 42–58. doi: 10.1080/07908319908666568

[ref5] Català A. B. (2015) The cultural component in the first language (L1) teaching: cultural heritage, identity and motivation in language learning, In 15th International Conference of the Spanish Association of Language and Literature Education, 178, 20–25

[ref6] Chao C. (2022). Being a YouTuber that language learners recognize: a study on constructing language teacher identities in social media community of practice. System 109:102860. doi: 10.1016/j.system.2022.102860

[ref7] Clément R. Norton B. (2021). Ethnolinguistic vitality, identity and power: investment in SLA. J. Lang. Soc. Psychol. 40, 154–171. doi: 10.1177/0261927X20966734

[ref8] Collie P. Kindon S. Liu J. Podsiadlowski A. (2010). Mindful identity negotiations: the acculturation of young Assyrian women in New Zealand. Int. J. Intercult. Relat. 34, 208–220. doi: 10.1016/j.ijintrel.2009.08.002

[ref9] Darvin R. (2017). Social class and the inequality of English speakers in a globalized world. J English Lingua Franca 6, 287–311. doi: 10.1515/jelf-2017-0014

[ref10] Darvin R. Norton B. (2015). Identity and a model of Investment in applied linguistics. Annu. Rev. Appl. Linguist. 35, 36–56. doi: 10.1017/S0267190514000191

[ref11] Darvin R. Norton B. (2021). Investment and motivation in language learning: What’s the difference? Lang. Teach. 56, 29–40. doi: 10.1017/S0261444821000057

[ref12] Dervin F. (2016). “Misnomers” in Interculturality in education: a theoretical and methodological toolbox. ed. Dervin F. (London: Palgrave Macmillan UK), 7–22.

[ref13] Desselle S. P. Clubbs B. H. Darbishire P. L. (2023). Motivating language and social provisions in the inculcation of pharmacy students’ professional identity. Am. J. Pharmac. Educ. 87:100010. doi: 10.1016/j.ajpe.2022.11.002, PMID: 37316120

[ref14] Döring M. Zunino F. (2014). Nature cultures in old and new worlds. Steps towards an ecolinguistic perspective on framing a “new” continent. Lang. Sci. 41, 34–40. doi: 10.1016/j.langsci.2013.08.005

[ref15] Dorjee T. Baig N. Ting-Toomey S. (2013). A social ecological perspective on understanding “honor killing”: an intercultural moral dilemma. J. Intercult. Commun. Res. 42, 1–21. doi: 10.1080/17475759.2012.723024

[ref46] Dorjee T. Ting-Toomey S. (2020). “Understanding intergroup conflict complexity: an application of the socioecological framework and the integrative identity negotiation theory,” Negot. Confl. Manag. Res. 13, 244–262. doi: 10.1111/ncmr.12190

[ref16] Downes S. (2001). Sense of Japanese cultural identity within an English partial immersion Programme: should parents worry? Int. J. Biling. Educ. Biling. 4, 165–180. doi: 10.1080/13670050108667726

[ref17] Duronto P. M. Nishida T. Nakayama S. (2005). Uncertainty, anxiety, and avoidance in communication with strangers. Int. J. Intercult. Relat. 29, 549–560. doi: 10.1016/j.ijintrel.2005.08.003

[ref18] Edwards E. Burns A. (2016). Language teacher-researcher identity negotiation: an ecological perspective. TESOL Q. 50, 735–745. doi: 10.1002/tesq.313

[ref19] English F. Marr T. (2015). Why do linguistics? Reflective linguistics and the study of language. Bloomsbury Academic: London

[ref20] Farsiu S. (2021). An ecolinguistic perspective on Assyrian-Iranian migrants’ portrayal of emotions toward their linguistic resources. Lang. Sci. 83:101331. doi: 10.1016/j.langsci.2020.101331

[ref21] Finke P. (2017). “Transdisciplinary linguistics,” in The Routledge Handbook of Ecolinguistics, eds. Fill A. Penz H. (New York: Routledge) doi: 10.4324/9781315687391.ch27

[ref22] Gao F. (2021). Negotiation of native linguistic ideology and cultural identities in English learning: a cultural schema perspective. J. Multiling. Multicult. Dev. 42, 551–564. doi: 10.1080/01434632.2020.1857389

[ref23] Goulah J. (2017). Climate change and TESOL: language, literacies, and the creation of eco-ethical consciousness. TESOL Q. 51, 90–114. doi: 10.1002/tesq.277

[ref26] Gudykunst W. B. (1998). Applying anxiety\uncertainty management (AUM) theory to intercultural adjustment training. Int. J. Intercult. Relat. 22, 227–250. doi: 10.1016/S0147-1767(98)00005-4

[ref28] Gudykunst W. B. Nishida T. (2001). Anxiety, uncertainty, and perceived effectiveness of communication across relationships and cultures. Int. J. Intercult. Relat. 25, 55–71. doi: 10.1016/S0147-1767(00)00042-0

[ref25] Gudykunst W. B. (1995). “Anxiety/uncertainty management (AUM) theory: development and current status” in Intercultural communication theory. ed. Wiseman R. L. (Thousand Oaks, CA: Sage)

[ref24] Gudykunst W. B. (1993). “Toward a theory of effective interpersonal and intergroup communication: an anxiety/uncertainty management perspective” in Intercultural communication competence. eds. Wiseman R. L. Koester J. (Newbury Park, CA: Sage)

[ref27] Gudykunst W. B. (2005). “An anxiety/uncertainty management (AUM) theory of sojourner adjustment” in Theorizing about intercultural communication. ed. Gudykunst W. B. (Thousand Oaks, CA: Sage)

[ref29] Haiyan H. (2014). EFL learners study on cultural identity anxiety in Chinese context. PhD dissertation, Shanghai International Studies University.

[ref30] Hall S. (1996). Introduction: who needs “identity”?, In Hall S. Gay P. du (Ed.) Questions of cultural identity. London: SAGE, 1–17

[ref31] Haugen E. (2001). “The ecology of language” in The Ecolinguistics reader: language, ecology and environment. eds. Fill A. Mühlhäusler P. (London: Continuum), 57–66.

[ref32] Hoffmann C. (1995). Monolingualism, bilingualism, cultural pluralism and national identity: twenty years of language planning in contemporary Spain. Curr Issues Lang. Soc. 2, 59–90. doi: 10.1080/13520529509615435

[ref33] Hua J. Zheng L. Zhang G. Fan J. (2019). Proactive personality and cross-cultural adjustment: a moderated mediation model. Int. J. Intercult. Relat. 72, 36–44. doi: 10.1016/j.ijintrel.2019.06.003

[ref34] Huang G. Zhao R. (2021). Harmonious discourse analysis: approaching peoples’ problems in a Chinese context. Lang. Sci. 85:101365. doi: 10.1016/j.langsci.2021.101365

[ref35] Hullett C. R. Witte K. (2001). Predicting intercultural adaptation and isolation: using the extended parallel process model to test anxiety/uncertainty management theory. Int. J. Intercult. Relat. 25, 125–139. doi: 10.1016/S0147-1767(00)00047-X

[ref36] Kaushal S. Dhammi S. Guha A. (2022). Climate crisis and language – a constructivist ecolinguistic approach. Materials Today 49, 3581–3584. doi: 10.1016/j.matpr.2021.08.093

[ref37] Kilanowski J. F. (2017). Breadth of the socio-ecological model. J. Agromedicine 22, 295–297. doi: 10.1080/1059924X.2017.1358971, PMID: 28742433

[ref38] Kim Y. Y. (2001) Becoming intercultural: An integrative theory of communication and cross-cultural adaptation. Sage: Thousand Oaks, CA

[ref39] Kim Y. Y. (2015). Finding a “home” beyond culture: The emergence of intercultural personhood in the globalizing world. Int. J. Intercult. Relat. 46, 3–12. doi: 10.1016/j.ijintrel.2015.03.018

[ref40] Kim Y. Y. (2017a). “Identity and intercultural communication,” in The international encyclopedia of intercultural communication, ed. Kim Y. Y. (New York: John Wiley & Sons), 1–9. doi: 10.1002/9781118783665.ieicc0002

[ref41] Kim Y. Y. (2017b). “Integrative communication theory of cross-cultural adaptation,” in The international encyclopedia of intercultural communication, ed. Kim Y. Y. (New York: John Wiley & Sons), 1–13. doi: 10.1002/9781118783665.ieicc0041

[ref42] Kim Y. Y. (2017c). “Stress–adaptation–growth dynamic,” in The international encyclopedia of intercultural communication, ed. Kim Y. Y. (New York: John Wiley & Sons), 1–6. doi: 10.1002/9781118783665.ieicc0071

[ref43] Komisarof A. Leong C.-H. Lim T. (2023). Constructions of Japanese national identity: host views using a social markers of acceptance framework. Int. J. Intercult. Relat. 94:101806. doi: 10.1016/j.ijintrel.2023.101806

[ref44] Kozaki Y. Ross S. J. (2011). Contextual dynamics in foreign language learning motivation. Lang. Learn. 61, 1328–1354. doi: 10.1111/j.1467-9922.2011.00638.x

[ref45] Kramsch C. Vork Steffensen S. (2008). “Ecological perspectives on second language acquisition and socialization” in Encyclopedia of language and education. ed. Hornberger N. H. (Boston, MA: Springer US), 2595–2606.

[ref47] Kung F.-W. (2016). The reorientation of international expatriates’ language and cultural identities from a religious ethnic community institution and community of practice: a multicultural and multilingual perspective. Lang Intercult Commun 16, 535–551. Available at:. doi: 10.1080/14708477.2016.1158830

[ref48] Lai M. L. (2011). Cultural identity and language attitudes – into the second decade of postcolonial Hong Kong. J. Multiling. Multicult. Dev. 32, 249–264. doi: 10.1080/01434632.2010.539692

[ref49] Lapresta C. Huguet Á. (2008). A model of relationship between collective identity and language in pluricultural and plurilingual settings: influence on intercultural relations. Int. J. Intercult. Relat. 32, 260–281. doi: 10.1016/j.ijintrel.2007.10.004

[ref50] Leather J. Dam J. V. (2003) Towards an ecology of language acquisition, In Leather J. Dam J. van (Eds) Ecology of language acquisition. Dordrecht: Springer Netherlands, 1–29

[ref51] Li J. Steffensen S. V. Huang G. (2020). Rethinking ecolinguistics from a distributed language perspective. Lang. Sci. 80:101277. doi: 10.1016/j.langsci.2020.101277

[ref53] Liu F. Chang C. (2021). Constructing a national identity in media editorials to promote affiliation with an international readership. Discourse Context Media 43:100538. Available at:. doi: 10.1016/j.dcm.2021.100538

[ref52] Li Y. Sun J. Zhu L. (2023). Ethnic elements in Chinese cosmetic brands: an exploration of digital communication effects on the recognition of Chinese national identity. Telemat Inform Reports 10:100046. doi: 10.1016/j.teler.2023.100046

[ref54] Logan S. Steel Z. Hunt C. (2016). Intercultural willingness to communicate within health services: investigating anxiety, uncertainty, ethnocentrism and help seeking behaviour. Int. J. Intercult. Relat. 54, 77–86. doi: 10.1016/j.ijintrel.2016.07.007

[ref55] Lou N. M. (2021). Acculturation in a postcolonial context: language, identity, cultural adaptation, and academic achievement of Macao students in mainland China. Int. J. Intercult. Relat. 85, 213–225. doi: 10.1016/j.ijintrel.2021.10.004

[ref56] Lund B. D. Walston M. (2020). Anxiety-uncertainty management theory as a prelude to Mellon’s library anxiety. J. Acad. Librariansh. 46:102160. doi: 10.1016/j.acalib.2020.102160

[ref57] Lynch R. Motha S. (2023). Epistemological entanglements: decolonizing understandings of identity and knowledge in English language teaching. Int. J. Educ. Res. 118:102118. doi: 10.1016/j.ijer.2022.102118

[ref58] Macias H. (2023). Language as a marker of cultural identity and commodification: the language socialization practices of multilingual, Latina/Mexican American mothers. Crit. Inq. Lang. Stud. 20, 77–104. doi: 10.1080/15427587.2022.2102010

[ref59] Malhotra G. Ramalingam M. (2022). Does impact of campaign and consumer guilt help in exploring the role of national identity and purchase decisions of consumers? J. Retail. Consum. Serv. 65:102839. doi: 10.1016/j.jretconser.2021.102839

[ref60] Martinez L. V. Ting-Toomey S. Dorjee T. (2016). Identity management and relational culture in interfaith marital communication in a United States context: a qualitative study. J. Intercult. Commun. Res. 45, 503–525. doi: 10.1080/17475759.2016.1237984

[ref61] Matveev A. (2017). “Theoretical foundations of intercultural competence” in Intercultural competence in organizations: A guide for leaders, educators and team players. ed. Matveev A. (Cham: Springer International Publishing), 27–48.

[ref62] Miller A. N. Samp J. A. (2007). Planning intercultural interaction: extending anxiety/uncertainty management theory. Commun. Res. Rep. 24, 87–95. doi: 10.1080/08824090701304717

[ref63] Monscheuer O. (2023). National identity and the integration of second-generation immigrants. Labour Econ. 82:102327. doi: 10.1016/j.labeco.2023.102327

[ref64] Nash J. Mühlhäusler P. (2014). Linking language and the environment: the case of Norf’k and Norfolk Island. Lang. Sci. 41, 26–33. doi: 10.1016/j.langsci.2013.08.004

[ref65] Ni L. Wang Q. (2011). Anxiety and uncertainty Management in an Intercultural Setting: the impact on organization–public relationships. J. Public Relat. Res. 23, 269–301. doi: 10.1080/1062726X.2011.582205

[ref66] Nordstrom J. (2020). Teaching in the periphery: teacher identity in community language schools. Teach. Teach. Educ. 96:103192. doi: 10.1016/j.tate.2020.103192

[ref9001] Norton B. (2013). Identity and language learning: extending the conversation. 2nd ed. Bristol, UK: Multilingual Matters. 43–45.

[ref67] Norton B. (1995). Social identity, investment, and language learning. TESOL Q. 29, 9–31. doi: 10.2307/3587803

[ref68] Norton B. De Costa P. I. (2018). Research tasks on identity in language learning and teaching. Lang. Teach. 51, 90–112. doi: 10.1017/S0261444817000325

[ref69] Norton C. Hulme M. (2019). Telling one story, or many? An ecolinguistic analysis of climate change stories in UK national newspaper editorials. Geoforum 104, 114–136. doi: 10.1016/j.geoforum.2019.01.017

[ref9002] Oetzel J. G. Ting-Toomey S. Rinderle S. (2006). “Conflict communication in contexts: A social ecological perspective,” in The Sage handbook of conflict communication, Eds. Oetzel J. G. Ting-Toomey S., SAGE Publications, Inc. 727–739. doi: 10.4135/9781412976176

[ref70] Oetzel J. Ting-Toomey S. Anderson W. J. (2013) “Conflict communication in contexts: Organizing themes and future directions,” in The SAGE Handbook of Conflict Communication, 2nd ed. Eds, Oetzel J. Ting-Toomey Anderson W. (Thousand Oaks: SAGE Publications), doi: 10.4135/9781452281988.n35

[ref71] Pang H. (2020). Is active social media involvement associated with cross-culture adaption and academic integration among boundary-crossing students? Int. J. Intercult. Relat. 79, 71–81. doi: 10.1016/j.ijintrel.2020.08.005

[ref72] Pehrson S. Brown R. Zagefka H. (2009). When does national identification lead to the rejection of immigrants? Cross-sectional and longitudinal evidence for the role of essentialist in-group definitions. Br. J. Soc. Psychol. 48, 61–76. doi: 10.1348/014466608X288827 18302807

[ref73] Peng A. Patterson M. M. (2022). Relations among cultural identity, motivation for language learning, and perceived English language proficiency for international students in the United States. Lang. Cult. Curric. 35, 67–82. doi: 10.1080/07908318.2021.1938106

[ref74] Peng L. Zheng Q. Zhang J. (2019). Multicultural and colorblind, investigating intergroup ideologies and relevant relations in mainland China. Int. J. Intercult. Relat. 70, 1–6. doi: 10.1016/j.ijintrel.2019.02.005

[ref75] Peng R.-Z. Zhu C. Wu W.-P. (2020). Visualizing the knowledge domain of intercultural competence research: a bibliometric analysis. Int. J. Intercult. Relat. 74, 58–68. doi: 10.1016/j.ijintrel.2019.10.008

[ref76] Pérez I. C. (2015). Indigenous languages, identity a nd legal framework in Latin America: an ecolinguistic approach1. Procedia. Soc. Behav. Sci. 212, 111–116. doi: 10.1016/j.sbspro.2015.11.307

[ref77] Petreñas C. Lapresta C. Huguet Á. (2018). Redefining cultural identity through language in young Romanian migrants in Spain. Lang. Intercult. Commun. 18, 225–240. doi: 10.1080/14708477.2016.1221416

[ref78] Pidduck R. J. Shaffer M. A. Zhang Y. Cheung S. S. Y. Yunlu D. G. (2022). Cultural intelligence: an identity lens on the influence of cross-cultural experience. J. Int. Manag. 28:100928. doi: 10.1016/j.intman.2022.100928

[ref79] Prince A. G. (2021). Managing anxiety and uncertainty: applying anxiety/uncertainty management theory to university health professionals and students’ communication. J. Commun. Healthc. 14, 293–302. doi: 10.1080/17538068.2021.1913946

[ref80] Qumrul Hasan Chowdhury (2016). “Construction of heritage language and cultural identities,” in The Routledge Handbook of Language and Identity, ed. Siân P. (New York: Routledge), 476–492. doi: 10.4324/9781315669816.ch30

[ref81] Rentschler R. Fillis I. Lee B. (2023). National identity and the future of branding the arts. Futures 145:103078. doi: 10.1016/j.futures.2022.103078

[ref82] Robertson W. B. Yazan B. (2022). Navigating tensions and asserting agency in language teacher identity: a case study of a graduate teaching assistant. Linguist. Educ. 71:101079. doi: 10.1016/j.linged.2022.101079

[ref83] Salmon-Letelier M. (2022). The shaping of national identity in diverse Nigerian secondary schools. Int. J. Educ. Dev. 89:102540. doi: 10.1016/j.ijedudev.2021.102540

[ref84] Samochowiec J. Florack A. (2010). Intercultural contact under uncertainty: the impact of predictability and anxiety on the willingness to interact with a member from an unknown cultural group. Int. J. Intercult. Relat. 34, 507–515. doi: 10.1016/j.ijintrel.2010.05.003

[ref85] Sang Y. (2023). Uncovering language socialization mechanisms in language teacher identity formation: an ethnographic study in a Chinese culture class. Linguist. Educ. 73:101138. doi: 10.1016/j.linged.2022.101138

[ref86] Sarmiento A. V. Pérez M. V. Bustos C. Hidalgo J. P. del Solar J. I. V. (2019). Inclusion profile of theoretical frameworks on the study of sociocultural adaptation of international university students. Int. J. Intercult. Relat. 70, 19–41. doi: 10.1016/j.ijintrel.2019.02.004

[ref87] Schecter S. R. (2014). “Language, culture, and identity” in The Routledge Handbook of Language and Culture, ed. Sharifian F. (New York, NY: Routledge), 3–18. doi: 10.4324/9781315793993.ch14

[ref88] Skjeggestad E. Gerwing J. Gulbrandsen P. (2017). Language barriers and professional identity: a qualitative interview study of newly employed international medical doctors and Norwegian colleagues. Patient Educ. Couns. 100, 1466–1472. doi: 10.1016/j.pec.2017.03.007, PMID: 28283216

[ref89] Soldatova G. Geer M. (2013). ‘“Glocal” Identity, Cultural Intelligence and Language Fluency’, Procedia – Soc. Behav. Sci. 86, 469–474. doi: 10.1016/j.sbspro.2013.08.599

[ref90] Steffensen S. V. Cowley S. J. (2021). “Thinking on behalf of the world: radical embodied ecolinguistics,” in The Routledge Handbook of Cognitive Linguistics, Eds. Wen X. Taylor J. R. (New York: Routledge. 723–736. doi: 10.4324/9781351034708-47)

[ref91] Steffensen S. V. Fill A. (2014). Ecolinguistics: the state of the art and future horizons. Lang. Sci. 41, 6–25. doi: 10.1016/j.langsci.2013.08.003

[ref92] Steffensen S. V. Kramsch C. (2017). “The ecology of second language acquisition and socialization” in Language Socialization. eds. Duff P. A. May S. (Cham: Springer International Publishing), 17–32.

[ref93] Stibbe A. (2021) Ecolinguistics: Language, Ecology and the Stories We Live By. Routledge: London and New York

[ref9004] Sussman N. M. (2000). The dynamic nature of cultural identity throughout cultural transitions: why home is not so sweet, Pers. Soc. Psychol. Rev. 4, 355–373. doi: 10.1207/S15327957PSPR0404_5

[ref94] Ting-Toomey S. (2007). Intercultural conflict training: theory-practice approaches and research challenges. J. Intercult. Commun. Res. 36, 255–271. doi: 10.1080/17475750701737199

[ref95] Ting-Toomey S. (2010). Applying dimensional values in understanding intercultural communication. Commun. Monogr. 77, 169–180. doi: 10.1080/03637751003790428

[ref96] Ting-Toomey S. (2015). “Identity negotiation theory,” in The International Encyclopedia of Interpersonal Communication, ed. Kim Y. Y. (New York: John Wiley & Sons), 1–10. doi: 10.1002/9781118540190.wbeic129

[ref97] Ting-Toomey S. (2017). “Identity negotiation theory,” in The International Encyclopedia of Intercultural Communication, ed. Kim Y. Y. (New York: John Wiley & Sons), 1–6. doi: 10.1002/9781118783665.ieicc0039

[ref98] Toomey A. Dorjee T. Ting-Toomey S. (2013). Bicultural identity negotiation, conflicts, and intergroup communication strategies. J. Intercult. Commun. Res. 42, 112–134. doi: 10.1080/17475759.2013.785973

[ref99] Torop P. (2014). “Cultural Semiotics,” in The Routledge Handbook of Language and Culture, ed. Sharifian F. (New York: Routledge), 170–181. doi: 10.4324/9781315793993.ch12

[ref100] Uryu M. Steffensen S. V. Kramsch C. (2014). The ecology of intercultural interaction: timescales, temporal ranges and identity dynamics. Ecolinguistics 41, 41–59. doi: 10.1016/j.langsci.2013.08.006

[ref101] Wood B. E. (2022). Belonging to the nation: negotiating narratives of national identity in Aotearoa New Zealand. Polit. Geogr. 99:102790. doi: 10.1016/j.polgeo.2022.102790

[ref102] Zahoor M. Janjua F. (2020). Green contents in English language textbooks in Pakistan: an ecolinguistic and ecopedagogical appraisal. Br. Educ. Res. J. 46, 321–338. doi: 10.1002/berj.3579

[ref103] Zhang L. Hwang Y. (2023). “Should I change myself or not?”: examining (re)constructed language teacher identity during the COVID-19 pandemic through text-mining. Teach. Teach. Educ. 127:104092. doi: 10.1016/j.tate.2023.104092, PMID: 36911756 PMC9988713

[ref104] Zhang Y. Wang J. Zhang L. (2023). Tourists’ national identity at heritage sites of natural disasters. J. Hosp. Tour. Manag. 55, 282–291. doi: 10.1016/j.jhtm.2023.04.007

[ref105] Zhou W. (2017). Ecolinguistics: towards a new harmony. Lang. Sci. 62, 124–138. doi: 10.1016/j.langsci.2017.04.004

